# The Roles of Immune Memory and Aging in Protective Immunity and Endogenous Reactivation of Tuberculosis

**DOI:** 10.1371/journal.pone.0060425

**Published:** 2013-04-08

**Authors:** Giorgio Guzzetta, Denise Kirschner

**Affiliations:** 1 Department of Statistics and Mathematics Applied to Economics, University of Pisa, Pisa, Italy; 2 Fondazione Bruno Kessler, Trento, Italy; 3 Department of Microbiology and Immunology, University of Michigan Medical School, Ann Arbor, Michigan, United States of America; Albert Einstein College of Medicine, United States of America

## Abstract

Finding more effective vaccines against tuberculosis (TB) and improved preventive treatments against endogenous reactivation of latent TB is strategic to block transmission and reach the WHO goal of eliminating TB by 2050. Key related open questions in TB research include: i) what are the determinants of a strong memory response upon primary infection? ii) what is the role of cytokines towards protective memory response against a secondary infection? iii) what are the mechanisms responsible for the increased risk of reactivation in elderly individuals? To address these questions, we explored a computational model of the immune response to *Mycobacterium tuberculosis* including a mathematical description of immunosenescence and the generation and maintenance of immune memory. Sensitivity analysis techniques, together with extensive model characterization and *in silico* experiments, were applied to identify key mechanisms controlling TB reactivation and immunological memory. Key findings of this study are summarized by the following model predictions: i) increased strength and duration of memory protection is associated with higher levels of Tumor Necrosis Factor-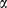 (TNF) during primary infection; ii) production of TNF, but not of interferon-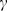, by memory T cells during secondary infection is a major determinant of effective protection; iii) impaired recruitment of CD4+ T cells may promote reactivation of latent TB infections in aging hosts. This study is a first attempt to consider the immune dynamics of a persistent infection throughout the lifetime of the host, taking into account immunosenescence and memory. While the model is TB specific, the results are applicable to other persistent bacterial infections and can aid in the development, evaluation and refinement of TB treatment and/or vaccine protocols.

## Introduction

Tuberculosis (TB), mediated by the airborne pathogen *Mycobacterium tuberculosis* (Mtb), is a major global health concern, with an estimated 9 million new cases and 1.5 million deaths worldwide each year [1]. To meet the World Health Organization’s (WHO) objective of eliminating tuberculosis by 2050, improved vaccines and treatments are needed [2]. The only vaccine currently licensed against Mtb (Bacillus Calmette-Guerin, BCG) dates back to 1921 and is not able to induce herd immunity in a population [3] due to its limited efficacy and duration of induced immunity [4]. In addition to the burden of disease, about one third of the world population is asymptomatically infected with latent Mtb [1]. A portion of this population will progress to clinical TB via endogenous reactivation of the latent infection. Thus, there is an enormous reservoir of potential sources of TB transmission (2 billion people), and therefore prevention of endogenous reactivation in high risk subjects (e.g., elderly, HIV positive or malnourished individuals, patients undergoing anti-TNF therapy) is strategic for control of global disease burden.

The development of new vaccines and treatments may be greatly aided by a more comprehensive understanding of the human immune response to Mtb. In this paper, we aim at identifying key mechanisms of protective immune memory against infection, and of endogenous reactivation in ageing individuals. To do so, we develop a computational model of the host-pathogen interactions taking into account events occurring during the entire lifetime of a host. Computational modeling has been successful in helping to elucidate the dynamics of the human immune response to Mtb [5], [6], [7], [8], [9] as well as to other persistent infections such as HIV-1 [10], [11], [12], Hepatitis C [13], [14], VZV [15] and H. pylori [16], [17], [18]. However, in none of these studies were the issues of memory or immunosenescence explored. Some important work has been done to describe the cellular dynamics of memory in response to infection with Lymphocytic Choriomeningitis Virus (LCMV), but these studies did not consider their effects during long-term persistent infections [19], . Mtb can be used as a model system for persistence, allowing us to extend some results of this study to other persistent infections. In the following paragraphs we provide an overview of current knowledge on immune memory generation and maintenance and immunosenescence, and on the host-Mtb immune dynamics in relation with them.

### Generation and Maintenance of Immune Memory

Upon infection with pathogens that cannot be cleared by the inflammatory response, an adaptive immune response is mounted, initially characterized by rapid clonal expansion of effector T cells (acute response phase). Once the immune system has succeeded in controlling the pathogen, a contraction phase follows where over 90% of effector cell are eliminated by apoptosis. The remainder fraction differentiates into a memory T cell phenotype [23]. Memory lymphocytes can be categorized in two functional phenotypes: effector and central memory T cells [24], [25]. Effector cells are mostly present in the blood and non-lymphoid tissues and can mount a rapid response to presentation of the same antigen they have previously seen through production of effector molecules (cytokines). Central cells reside in lymphoid organs and are comparatively slower in responding to antigen stimulation; however, they can switch to the effector memory phenotype [26]. Memory T cells of both types initially generated at the site of infection circulate in blood; those with a central memory phenotype can migrate to lymph nodes. We will refer to this compartment as *circulating memory T cells*. Circulating memory T cells are maintained over time through proliferation and homeostatic mechanisms; in the absence of re-infection, their concentration is slowly diluted over time due to thymic output of naive T cells and competition with memory T cells specific to other antigens [27], [19], [28]. Memory T cells are also found in the peripheral tissue (e.g., the lung airways) as *resident memory T cells* [29], where they play an important role for control of local infections by limiting pathogen replication in early phases of infection. Resident memory T cells have been shown to turnover rapidly and to be sustained by continuous recruitment from the circulating compartment [30].

Availability of memory T cells does not necessary imply protective immunity, i.e. that a new challenge from the same pathogen (secondary infection) will not result in an adverse outcome for the host. In fact, protective immunity stems from a combination of factors related to the ability of the immune system to effectively use memory T cells [19]. The duration of protective immunity, therefore, is not a linear function of the number of available T cells.

### Immunosenescence

Immunosenescence refers to the aging process in humans. This process of course occurs continuously in time for the lifespan of a host. Consequences of immunosenescence are the accumulation of changes in various components of the body, specifically including the immune system, often resulting in a reduced protection against exogenous pathogens [31]. Consequently, an increase in morbidity and mortality is found in elderly individuals for both acute and persistent infectious diseases [32]. The aging immune system is characterized by two key types of changes [33]: primary alterations, which appear as a direct effect of age on normal cell or tissue activity; and secondary alterations, which derive from attempts by the organism to compensate for these primary changes. For example, at the cell level, primary alterations manifest as reduced and/or slowed functionality (e.g., antigen processing and presentation, cell proliferation and activation and signaling pathways [34]), due to the accumulation of unrepaired mutations after DNA damage or oxidative stress [35]. At the organ level, the thymus undergoes involution with age, which results in reduced production of naive T cells and consequently an unbalance in the relative proportions of lymphocyte populations [36]. A lack of naive T cells reduces the capability of the immune system to amount an adaptive response to new infections [37]. The immune system reacts to thymic involution by a compensatory homeostatic expansion of circulating lymphocytes (secondary response), in order to maintain constant systemic levels of naive T cells [38]. However, in the long run homeostatic proliferation produces dysfunction due to cell aging [39]. These complex mechanisms are reviewed in [33], [34], [40].

Immunosenescence also has a detrimental effect on the memory response: while recall responses to infections acquired at young ages are not affected by the age of the host [33], age does impair generation of a new memory response in the elderly. In particular, memory T cells generated by an aging host function poorly in terms of cytokine production and proliferating capacity [41].

### Tuberculosis

The host-pathogen interactions between mycobacteria and humans are complex and only partially explained. When adequate contact between an infectious case and a susceptible host occurs, mycobacteria invade the host’s pulmonary alveoli, where adaptive immunity is activated. Invading Mtb are initially phagocytized by resting macrophages, where they are able to survive [42]. Infected macrophages secrete cytokines such as Tumor Necrosis Factor-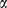 (TNF-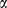) to recruit immune cells (mainly, CD4+ and CD8+ T cells) to the site of infection [43] and prime the activation of effector functions in these cells. In turn, cytokines secreted by T cells (primarily, Interferon-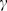 (IFN-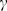) ) cause the activation of macrophages [44]. Bacteria are mainly killed by activated macrophages and by cytotoxic functions of activated CD4+ and CD8+ T cells, or by TNF-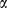 induced apoptosis of infected macrophages. Overall, the balance between pro-inflammatory and anti-inflammatory properties of the different cytokines regulates the balance between effectiveness of the immune response and local tissue damage and destruction due to inflammation. Contrary to traditional thinking which considers humoral immunity irrelevant [45], recent explorations have demonstrated a role also for B lymphocytes towards protection against mycobacterial infections [46]. However, the mechanisms and entity of this contribution have not been fully elucidated yet, so we will adhere to the classical theory throughout the rest of this paper.

At the end of the acute phase of the immune response, generally a few days to a few months from initial Mtb infection, three different outcomes are possible: complete removal of the pathogen (clearance); persistence of the pathogen that appears to be a balance between host and pathogen occurring within and constrained by structures that develop in lungs called granulomas (latent TB infection, or LTBI); and uncontrolled growth of bacteria with severe symptoms and extensive tissue destruction that usually causes host’s death if untreated (active disease). Eventually, it is the balance between bacterial replication and bactericidal activity of the host immune response that determines the clinical outcome for the host [43], [47].

Several published mathematical models have quantitatively elucidated many aspects of the human host’s immune response to Mtb challenge (e.g., [48], [49], [50], [7]). These models predict an evolution in time of immune variables that reproduces three main short-term outcomes (clearance, containment and disseminating infection). However, none of the available models can account for dynamics occurring at time scales beyond the acute phase of infection. In this study, we propose a model that is able to reproduce the lifelong dynamics of Mtb infection in a human host.

Over the lifetime of an individual other events related to Mtb infection occur that are important also from an epidemiological perspective. Endogenous reactivation of a LTBI may occur with ageing or other immunodeficiencies: in this case, infection within a granuloma is no longer controlled, bacterial growth accelerates, and active disease occurs [51]. Endogenous reactivation may occur even several decades after primary infection [52] and is by far the most common form of TB in high-resource, low-incidence countries [53]. Immunological studies and data suggest that impaired or delayed availability of TB-specific CD4+ cells at the site of infection is an important determinant of the increased susceptibility to primary TB disease in elderly [54] and HIV positive individuals [43], but mechanisms of TB reactivation have not been identified.

Another process that is relevant to Mtb infection is immune memory generated during primary infection, which may be protective against secondary Mtb infection. In fact, epidemiological studies estimated that individuals with LTBI may be at reduced risk of exogenous reinfection TB [55] (i.e. active disease from a secondary contact with an infectious case).

In the case of TB, specific resident memory T cells have been shown to be present in the lungs of individuals infected with Mtb [56] and have proven protective for active TB in mice [57], [58], [59]. In a recent study [59], it was found that the majority of TB-specific resident memory T cells are locally proliferating in mice, with only a smaller contribution deriving from recruitment of circulating memory T cells. Such local proliferation is antigen-dependent [59]. However, TB infection in mice is disseminated, therefore TB antigens are available at all places in the lungs. In human adults instead, TB infection is generally confined to granulomas, and therefore antigen-dependent proliferation of resident memory T cells at lung sites different than the granulomas is unlikely. Antigen presentation also occurs in the lymph nodes, but resident memory T cells do not have homing potential for the lymph node [25]. Therefore, we will assume that TB-specific resident memory T cells in lung alveoli not involved in Mtb infection will only be sustained by T cell recruitment from the circulating compartment [29]. In addition this antigen-independent recruitment, which serves to replace dead resident memory T-cells, an antigen-dependent recruitment of circulating memory T cells has been also shown to take place for TB during secondary infection [58], [59], but its contribution was not found determinant for protection from exogenous reinfection.

## Materials and Methods

Our goal is to develop a mathematical model of the host-immune response to Mtb infection that includes the dynamics and long-term processes of aging and memory to explore their effects on disease progression and reactivation and on the outcome of secondary infection. To this end we present the model and analysis tools that we applied in this study.

### Reference Model

The model proposed in this work is an adaptation of a previously published Ordinary Differential Equations (ODE) model [50], accounting for the evolution in time of the numbers of macrophages, T cells and Mtb, and of the concentrations of cytokines responding to infection in the lung by Mtb. We tracked the rates of change of sub-species for each of these cell types (for example, resting, activated and infected macrophage cell types) and the non-linear interactions between cell types. We used this model to explore the role of CD8+ T cells in an integrative way, as their role had not yet been completely elucidated in TB [50]. As with all of our models, we included both published and unpublished data to build, test and validate our model. The model was then used to simulate virtual experimental situations, such as deletion of CD8+ T cell subsets. The model predicted a differential contribution for CD8+ effector T cells that are cytotoxic as compared with those that produce IFN-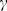. The model was also used to simulate a hypothetic vaccine and determined the minimum hypothetical levels of effector memory cells of each T cell subset (CD4+ and CD8+) necessary for protection. This model represents total lung tissue (as it is not a spatial model, for example, capturing events occurring in a granuloma). We believe considering a non-spatial model when examining the effects of aging and immunosenescence is an acceptable assumption as we are concerned with events occurring in total lung and not at the scale of a single granuloma. Thus, total lung can be considered a well mixed compartment regarding cells and bacteria. Thus, infection outcomes such as bacterial clearance, or uncontrolled growth of bacteria represent outcomes at the host scale.

To perform our studies herein, we modify this existing model [50] to include two main mechanisms: immunosenescence (aging), and the generation and maintenance of immune memory. We describe below how we capture these processes and how we incorporate them into our mathematical model. The equations of the reference model [50] are reported in Section S1 of File S1.

### Including Immunosenescence in the Model

An observed side-effect of immunosenescence on the response to Mtb infection is the impaired ability of the immune system to recruit TB-specific T-helper type 1 (Th1) cells to the site of infection, as shown in elderly subjects [54]. We hypothesize that this same mechanism may be responsible for both the increased susceptibility to primary active TB in older individuals [54], [55] and the endogenous reactivation of TB in latently infected hosts. In the reference model [50], two parameters (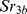 and 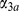) modulate the CD4+ T cells recruitment rate, dependent respectively on the concentration of TNF-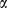 and of chemokines secreted by macrophages. To model aging, we replace these two constant parameters with decreasing functions dependent on person age. In particular, we assume for simplicity that both parameters follow the same evolution in time by means of a common aging function, named 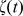:
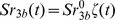


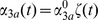
where 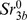 and 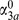 are the parameter values at birth, 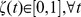 and 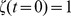.

Aging is a process of risk accumulation: for biological systems, this is suitably described by the Gompertz law [60], [61], [62], which assumes the risk of a breakdown event to increase exponentially with time. Here, we suggest to consider function 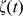 as inversely proportional to the risk of breakdown, since it represents the level of retained functionality of the parameter which it modulates. Based on these biological considerations, 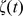 is assumed to take a negative exponential form with rate 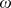:
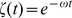
with 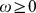 (years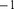).

**Figure 1:**
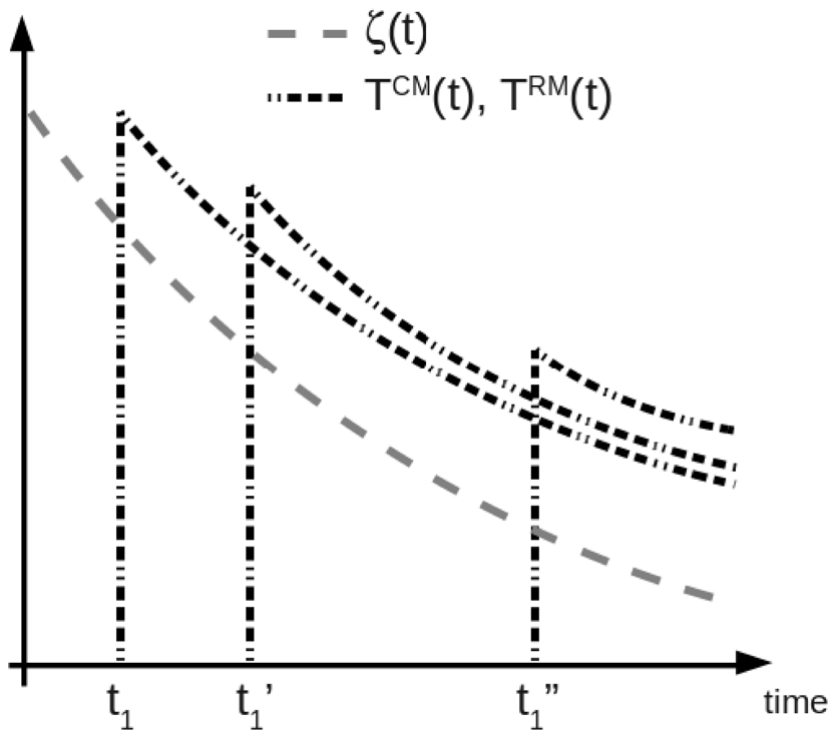
Features included in the proposed model. Immunosenescence is represented by the decrease in time (age of the host) of function 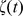, which drives the decline of CD4+ T cells recruitment. Memory generation is represented by the availability of memory T cells after primary infection (in quantities dependent on the time of primary infection, 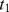 vs. 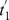 vs. 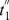). Memory maintenance is represented by the slow decline in time of the initial pool of memory T cell, which is due to mechanisms of immune memory dilution. The y axis has no meaning, since the curves only represent the qualitative behavior of 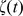, 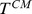 and 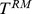. The latter two show the same qualitative pattern, but in quantitative terms 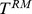 is 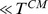. See section Materials and Methods for more details.

Function 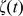 is depicted in Figure 1 as a dashed grey line. While there are no specific data to support a negative exponential form for the aging of T cell recruitment, previous experimental studies on related immunological variables, such as the generation of T cells in the thymus of aging humans [36], support the use of this functional form. According to this model and in the absence of memory, the outcome of infection with Mtb will depend on the host’s immune parameters, including 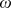, the number of inhaled bacteria (bacterial inoculum), 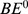 and the host’s age at which infection occurs 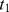, which determines the values of 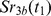 and 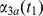 at infection initiation.

### Including Immune Memory Generation and Maintenance in the Model

**Figure 2:**
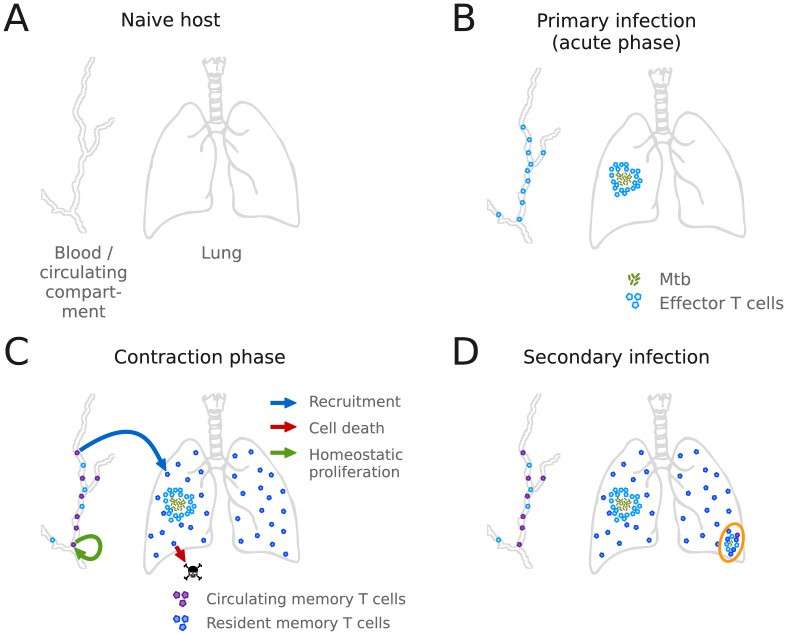
Conceptual framework of anatomical compartments and cell populations in the model of TB immune response with memory. (A) In a TB-unexperienced organism, both the lungs and the blood and lymph nodes are free of Mtb and TB-specific lymphocytes. (B) Upon primary Mtb infection, an adaptive response is mounted, and a large number of effector T cells is produced. (C) After stable containment of the pathogen in a granuloma is achieved, a memory response is mounted by the induction of memory T cells of both phenotypes; central memory T cells will be mostly present in the blood and lymph nodes, here termed *circulating compartment*, whereas effector memory T cells will be mostly present in the lung as resident memory T cells. Circulating memory T cells are maintained in time through homeostatic proliferation, and represent a recruitment reservoir for the turnover of dead resident memory T cells. (D) Upon secondary infection, resident memory T cells and additionally recruited circulating memory T cells help containing the pathogen in a more efficient way through a more rapid effector response.

The generation and maintenance of immune memory plays a key role in determining infection outcome when an individual previously infected with Mtb is challenged again with the same pathogen (called secondary infection). Typically, newly inhaled bacteria will colonize sites of the pulmonary alveoli which have not been previously colonized by Mtb from primary infection. A general framework for the dynamics of primary and secondary infection is presented in Figure 2. In a naive host (top left), no TB-specific T cells are available either in the lungs or in the blood and lymphoid tissues; when primary infection occurs (top right), during the acute phase effector T cells are generated in draining lymph nodes (LNs) and traffic between the lungs and LNs through blood and lymphatics. In an infection that becomes latent (bottom left), once the peak infection period ends and pathogen is contained in granulomas, memory T cells of both effector and central phenotypes are produced, of which some go into the circulating compartment, and some stay in lung as resident memory T cells. While both phenotypes can be present in both compartments, we assume all resident memory T cells to have an effector phenotype, and all circulating memory T cells to have a central phenotype [63]. When a secondary infection occurs (bottom right), available resident memory T cells plus circulating memory T cells recruited in an antigen-dependent fashion allow a more prompt, and possibly more effective, response than during primary infection.

The model from [50] is used to summarize immunological quantities at a given site in the lung and not to capture the spatial dynamics of granuloma formation (which is unfeasible using ODEs). This aspect of TB immunology has been explored in other works [8], [7], [49]) using a different modeling approach called Agent Based Modeling. To capture the dynamics of a secondary infection using ODEs, a new instance of the model is simulated assuming a new lung site, using the same initial conditions as we did for primary infection (see Table S1 in File S1), except that Th1 type CD4+ T cells and cytotoxic CD8+ T cells (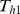 and 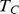 respectively), representing resident memory T cells, are initiated with non-zero values. When we simulated virtual vaccines in [50], these variables were assigned arbitrary non-zero values and different scenario simulations were performed. In this work, the numbers of resident memory T cells at the time of secondary infection 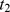 are calculated by explicitly modeling the dynamics of recruitment and turn-over of resident memory T cells (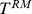) in time. The following equations are used, based on the model depicted in Figure 2, bottom left panel:
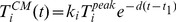
(1)

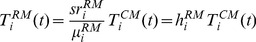
(2)


A mathematical derivation of these equations is reported in Section S2 of File S1. Hereafter we report the biological meaning of quantities involved:

- subscript 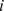 represents quantities and parameters related to CD4+ (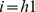) and CD8+ (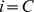) T cells respectively;

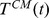
 is the concentration of circulating memory T cells (which are assumed to have a central phenotype) in the blood and lymphoid tissues at each time 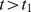;

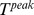
 is the peak number of effector T cells (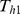 and 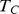) during the acute phase of primary infection;

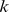
 is the fraction of effector cells which are initially transformed to circulating memory T cells, which we will term the *immunogenic potential* of the host against TB infection;

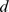
 is the rate at which the pool of circulating memory T cells wanes in time, according to mechanisms of immune memory dilution [19]; it is assumed to be the same for both CD4+ and CD8+ T cells.

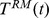
 is the number of resident memory T cells (which are assumed to have an effector phenotype) in the pulmonary alveoli at each time 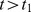;

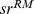
 is the rate at which 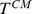 are recruited (antigen-independent) to the lungs and converted to an effector phenotype to replace the death of 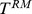;

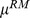
 is the death rate of resident memory T cells;

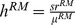
 is the rate at which circulating memory T cells turn over to serve as resident memory cells;

In order to account for the impaired memory generation by aging organisms, we force both parameters 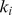 to be decreasing functions of person age, according to the same aging function assumed for 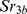 and 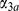:
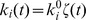
where 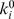 is the immunogenic potential (ability of the system to generate circulating memory T cells upon *Mtb* infection) at birth. We assume that circulating and resident memory T cells are instantaneously available from time 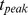, corresponding to the time at which both 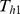 and 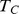 have reached their peak during primary infection. Dashed black lines in Figure 1 represent schematically the availability in time of either 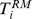 or 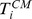 depending on different times of primary infection. Overall, Figure 1 summarizes how both the aging and memory features are introduced in the model.

The number of resident memory T cells evaluated at the time of secondary infection, 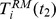, is used as initial condition for the levels of 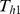 and 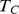 cells. Furthermore, an additive term representing antigen-dependent recruitment of circulating memory T cells and their conversion to the effector phenotype is considered in the model equations. The term contributes to the equations for both 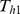 and 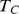, respectively, and has the form:
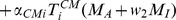
where 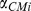 is the recruitment and conversion rate; 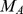 and 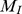 are the numbers of activated and infected macrophages, respectively, and represent the quantity of antigen available at the site of infection [50]; and 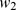 is a scaling coefficient. The form is based on the antigen-dependent recruitment of effector cells during acute primary infection.

### Parameter Range Estimation and Uncertainty and Sensitivity Analysis

Model [50] contains 103 parameters, whose ranges have been estimated from diverse datasets, theoretical considerations and calibrated simulations [48], [64], [9], [50]. The baseline values and ranges of these parameters are reported in Table S1 in File S1; unless otherwise specified, simulations are performed assigning baseline values to parameters.

Furthermore, the model proposed in this study includes 8 additional parameters, of which one is involved with representing immunosenescence and 7 others are used for modeling the generation and maintenance of immune memory. Table 1 reports ranges estimated for the new parameters and their baseline value; a derivation of these estimates (with references) is reported in Section S3 of File S1.

**Table 1:** Parameter values and ranges for immunosenescence and memory.

Parameter name	Symbol	Reference	Min	Max	Baseline	Unit
Aging rate	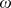	–	0.01	0.1	0.05	years^−1^
Immunogenic potential at birth for CD4	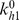	[63]	0.0625	0.25	0.1	ml^−1^
Immunogenic potential at birth for CD8	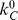	[73]	0.23	10	1.5	ml^−1^
Turnover rate of 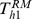	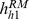	[56]	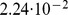	2	0.35	ml
Turnover rate of 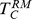	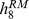	[56]	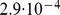	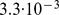	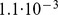	Ml
Rate of memory T cell dilution	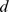	–	0.01	0.1	0.05	years^−1^
Additional recruitment rate for 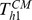	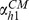	[63]	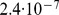	0.04	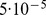	ml/Mph/day
Additional recruitment rate for 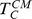	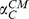	[73]	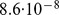	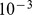	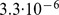	ml/Mph/day

However, the estimation of model parameters is highly uncertain due to lack of appropriate biological data and because it is difficult to verify assumptions regarding underlying processes. Therefore, statistical techniques are used to assess the sensitivity of model output to parameter uncertainty and the relative importance of modeled mechanisms associated to parameters. Uncertainty and sensitivity analysis is performed throughout the course of this study by Latin Hypercube Sampling (LHS) of the parameter space and calculation of Partial Rank Correlation Coefficients (PRCC) between parameters and outputs of interest. The procedure has been proven to be robust and efficient for models with a high number of parameters and high uncertainty [65]. If the absolute values of the PRCC calculated for a parameter-output pair is significantly greater (lower) than 0, then the mechanism represented by the given parameter plays a key positive (negative) role for the given output. Two target outputs were chosen for this study: i) the magnitude of immune memory at time 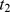, represented by numbers of CD4+ and CD8+ memory T cells in the circulating and resident compartments; and ii) an indicator of memory-induced protection against exogenous re-infection, termed 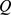. We define this quantity as the ratio of the bacterial loads at the end of secondary infection 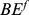 in two simulations, in one of which memory T cells at time 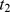 are forced to zero. All other parameters and times of infection 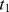 and 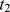 are equal. Differently from the number of memory T cells at 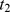, 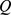 accounts for immune processes occurring after the onset of secondary infection. 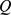 will be a number around 1 when the two models produce the same type of outcome: clearance (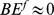 CFUs), LTBI (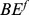 between a few units and a few thousands CFUs), or active disease (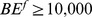 CFUs). 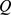 will be a number much larger than 1 if the memory model produces a more favorable outcome than the model without memory (i.e., clearance vs. latency, or latency vs. active disease); finally, 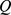 will be a very high number when the model without memory results in active disease and the memory model results in clearance. Therefore, when the outcome between memory and absence of memory is more favorable for the host, 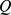 will assume higher values and therefore can be used as an indicator of memory-induced protection against exogenous re-infection.

### Knock-out Simulations

To analyze the relative effect of two different mechanisms of memory protection, we reproduce an “in silico” experiment in which the production of cytokines by resident memory T cells is blocked (a.k.a. “knocked out”) [58]. We do so by setting to zero from the start of the simulation the contribution of memory T cells (both CD4+ and CD8+ types) to the production of the considered cytokine while leaving unchanged that of effector T cells and all other factors.

## Results

The objective of this study is to investigate mechanisms that allow for a protective effect of immune memory, and those inducing endogenous reactivation, a major and problematic outcome of TB and more in general of persistent infections.

In this section, we present key findings from the study and validate the model. Because specific quantitative TB data is not available for many of the processes considered, we provide only qualitative assessment of the model outputs, showing a wide compliance of its predictions with published results from both human and animal TB experiments.

**Figure 3:**
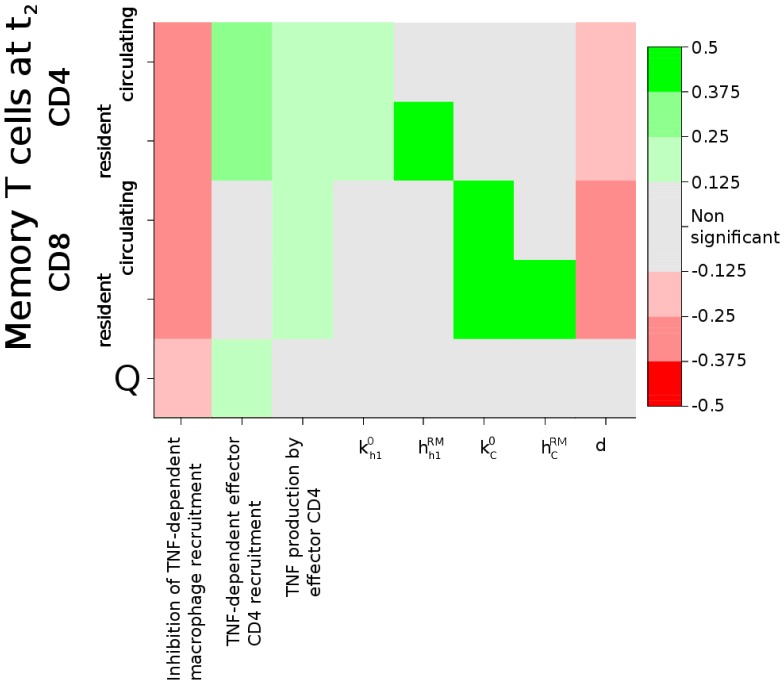
Results of the Uncertainty and Sensitivity analysis. PRCC values for all parameters resulted significant against the target variables. Grey blocks indicate non-significant correlation. Green blocks indicate positive correlation, red blocks negative correlation. Parameters which have not been introduced in the text are described verbally rather than with the corresponding symbol. 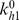: CD4+ specific immunogenic potential; 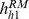: turnover rate of resident memory CD4+ T cells; 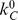: CD8+ specific immunogenic potential; 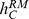: turnover rate of resident memory CD8+ T cells; 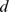: waning rate of circulating memory CD4+ and CD8+ T cells.

Some of the findings presented here were derived using the Uncertainty and Sensitivity analysis techniques described in Materials and Methods section. Besides providing a general assessment of the model parametrization, this technique allows us to identify the critical nodes of the system by evaluating the statistical effect of parameters variability on the desired output [65]. The outcome of this analysis is summarized in Figure 3 in terms of PRCC values for significant parameter/target pairs, and commented in detail throughout this section.

### Endogenous Reactivation

**Figure 4:**
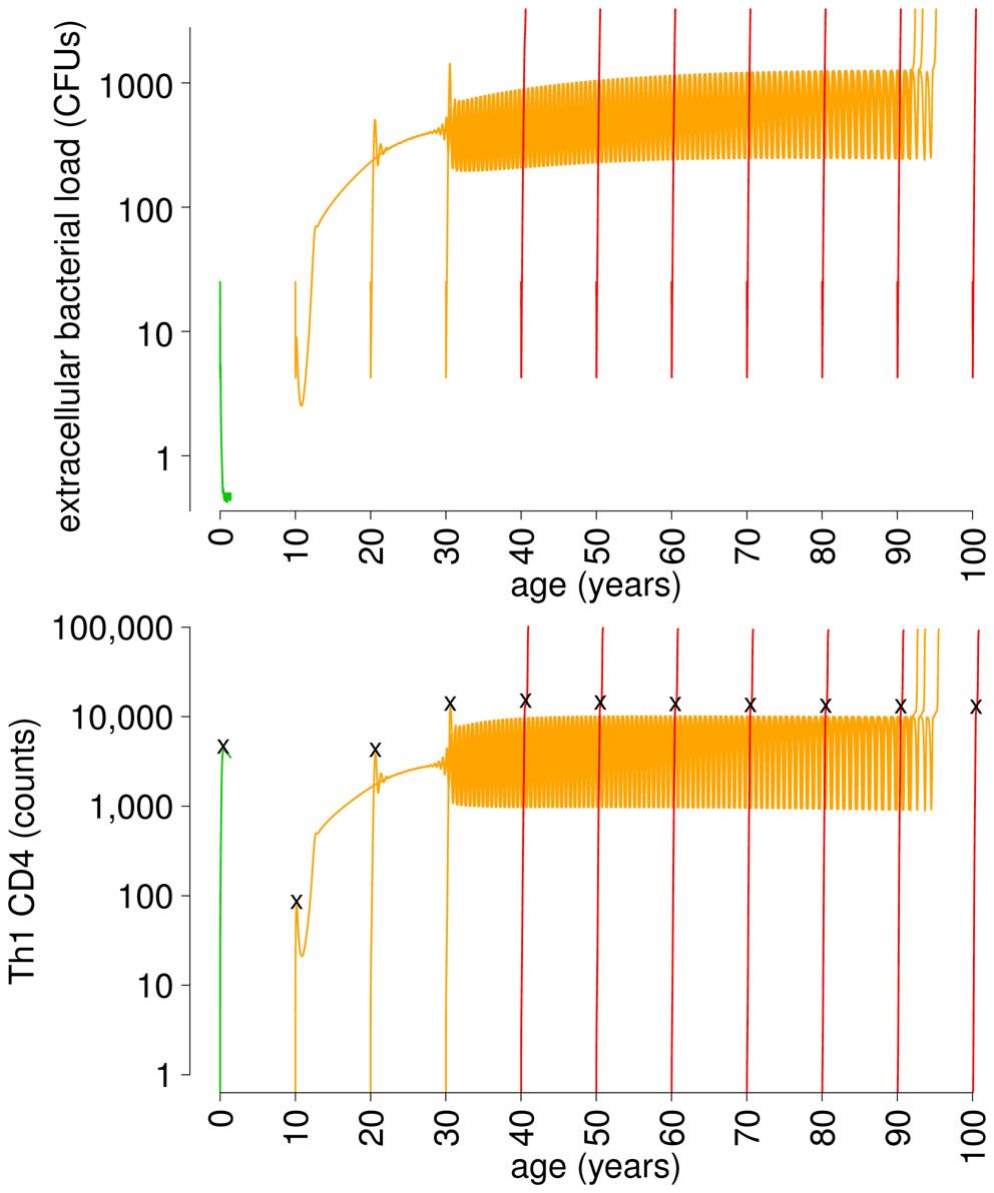
Endogenous reactivation. Reproduction of endogenous reactivation. The simulation is performed by using the same inoculum of 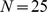 bacteria at initial times of primary infection 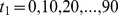 years and keeping the same set of immune parameters fixed (specified in Table S1 in File S1). Green: clearance of infection; orange: LTBI followed by reactivation; red: primary active disease. The oscillations in reactivation curves are not perfectly overlapping, but difference in oscillation phase are not visible at this time scale. In the lower panel, black marks indicate the number of T-helper type 1 (Th1) cells at the peak of the acute phase, a proportion of which is used to generate circulating memory T cells according to Equation 1. The number of T cells for infection at age 0 is only displayed until the time at which both extracellular and intracellular Mtb are cleared, after which the simulation is terminated.

Figure 4 summarizes one of the main results of this study, the model’s ability to predict endogenous reactivation of latent infections, while at the same time reproducing the increased probability of primary active TB observed in aged adults [55], [54]. Figure 4A shows examples of model outcomes for the same host (characterized by a given parameter set), experiencing primary infection occurring at different ages 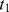. The simulated time course of the extracellular bacterial load (measured in colony forming units, CFUs) is displayed as a proxy for the qualitative outcome of infection, as we have done previously [50]. With the set of parameter values used for Figure 4, if the host is infected at age 0 years, the extracellular bacterial load is cleared rapidly (green line) (with different parameter values other outcomes are possible, including active disease or latent infection at age 0). If infection occurs at older ages (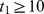 years in the specific case), the immune response is no longer capable of completely clearing Mtb during initial infection; however, bacterial load can be temporarily contained at low levels in an LTBI state (orange lines, until 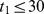 years). Later in life, around age 92–94, the latent infection breaks down into exponential bacterial growth, typically observed in disease, representing endogenous reactivation. Mtb can also grow uncontrolled just a few months after infection, representing progression to primary active disease (in this case infections occurring at age 40 or higher, red lines). Primary active disease is a feature predicted by previous short-term models of TB (e.g., [50]); however, the inclusion of aging in the model additionally allows to reproduce the increased risk of adult individuals to develop primary TB with respect to children, as estimated by classical epidemiological studies [55]. Even though Figure 4A refers to a specific set of parameter values, the model is able to reproduce endogenous reactivation in a broad region of the parameter space, as demonstrated in Section S4 of File S1.

Further insights on the system dynamics over time are obtained by looking at infections resulting in LTBI (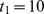 to 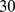 years). In this case, after the latent state has been established within a few months, the aging of the immune system perturbs the dynamical equilibrium: initially it allows a slow growth of bacteria, then it induces sustained oscillations and finally it causes endogenous reactivation. The trajectory of bacterial load during the acute phase of infection leading to the latent state is qualitatively different between infection occurring at 10 and 30 years. In the first case, the immune response almost clears the pathogen, while in the second case a large overshoot highlights the difficulty an aging immune system experiences when attempting to contain infection. After this time, however, the three simulations follow the same trajectories (except for possible phase shifts among oscillations). Most importantly, the model predicts that the age at which reactivation occurs does not depend significantly on when primary infection occurred.

A time course similar to bacterial load is followed by T cells. In Figure 4B we report counts for Th1 CD4+ T cells as an example of T cell population. T cell counts rise as the bacterial load increase, in order to improve control of the infection. Trajectories for bacterial load and T cells evolve with some delay with respect to one another, but it is not visible in the time scale used.

### Protective Immunity against Re-infection

To study the effect of immune memory, we need to consider at least two episodes of infection; the first (primary), occurring at time 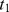, generates the pool of memory T cells; the second (secondary, or re-infection), occurring at time 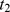, of which we want to consider the outcome with respect to the case where no memory is present. Such an outcome will not only depend on the level of immunosenescence of the host at time 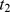, but also on the size of the pool of memory T cells, which in turn depends on the age of the host at primary infection 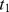.

**Figure 5:**
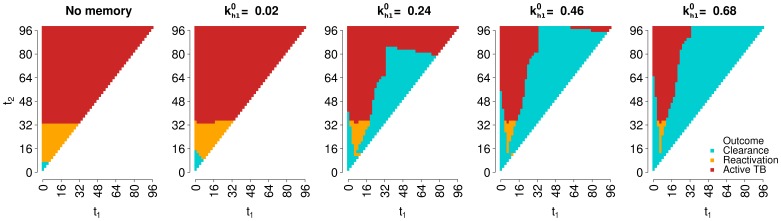
Characterization by CD4+ specific immunogenic potential, 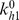. Outcomes of secondary infection for all possible combinations of time of primary infection 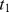 and time of secondary infection 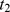 (constraining 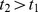), sampled with a step of 2 years (hence the pixel-like appearance of the graphs). All graphs in the figure have the same y axis, whose label is reported in the leftmost graph only. All parameters of the immune models are kept fixed at the same baseline values (see Table S1 in File S1), and the aging rate 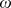 is set to 0.05 years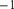; The CD4+ specific immunogenic potential 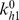 varies with the panels. Dark red: active disease; orange: LTBI followed by endogenous reactivation at some point of the host’s life; turquoise: clearance of primary infection.

Figure 5 shows the outcome of secondary infection for all possible ages of primary (x axis) and secondary (y axis) infection, at different values of parameter 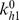, where superscript 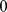 indicates the value of 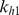 at birth. Panel A represents the case (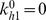) for which TB infection does not produce any immune memory. In this case the outcome of secondary infection does not depend on the time at which primary infection occurs, and regions of different outcomes are separated by horizontal lines. In other words, in the absence of memory the outcome of any secondary infection is exactly that of a primary infection occurring at that age. Panels B–E show that the areas of regions with a more favorable outcome for the host are progressively expanding with increasing values of parameter 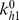, i.e. memory levels. In most cases, memory enables clearance of an infection that would otherwise result in active disease or lead to LTBI followed by reactivation. However, there are also cases for which memory prevents or delays the development of active disease by containing, at least temporarily, secondary infection.

Protection conferred by memory can be evaluated in terms of the differential outcome of a secondary infection at time 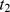 with respect to the outcome that the same infection would have if no memory were present: the absence of difference in these two outcomes is the sign of a waned protective immunity. Therefore, we define the duration of memory protection 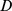 as the temporal distance in years between the time of primary infection 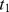 and the time 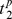 of the latest secondary infection for which memory is protective (hence superscript 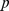 in 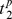).

**Figure 6:**
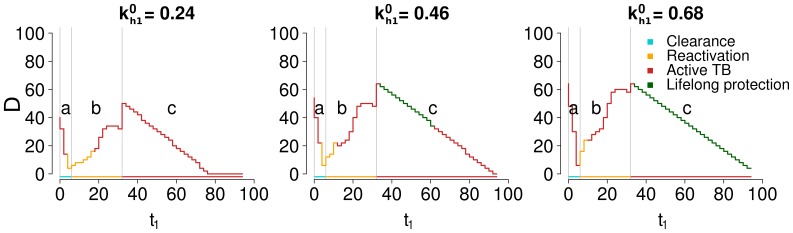
Duration of immunity D (years) by time of primary infection 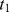 (years) and CD4+ specific immunogenic potential 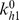 (ml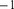). All graphs in the figure have the same y axis, whose label is reported in the leftmost graph only. In each panel, the color of the lines indicates the infection outcome for secondary infections occurring after memory protection has waned, i.e. at ages just above 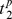 (age of the latest secondary infection for which memory is protective). If 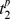 exceeds 100 years, the protection conferred by primary infection is considered lifelong. The curves are discrete because the times of primary and secondary infection have been sampled with a step of 2 years to reduce computational time.

Figure 6 shows the dependence of 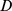 on the age of primary infection 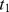 for different values of 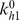. The horizontal line at the base of each panel shows the outcome of corresponding primary infection, and changes in primary outcome are marked by vertical grey lines. In all panels, a similar pattern is seen. For 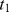 very low (a) the capacity of the immune system to generate immune memory (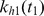) is high. However, due to the strength of immune response in younger individuals, primary infection results in clearance, which is characterized by a relatively low production of effector T cells. Therefore the two effects trade-off, producing a moderate duration of protection which falls rapidly with 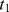 due to the decline of 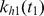. As 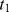 increases, the outcome of primary infection switches from clearance to LTBI (b). As shown in Figure 4B, the immune system has a growing difficulty in containing the infection in a latent state for increasing values of 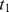. The number of effector T cells produced during the acute phase grows progressively with 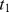 and so does the number of memory T cells produced, outweighing the opposite tendency due to the decline in 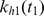; as a result 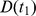 is also increasing in this region. When 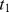 reaches a value so high that primary infection results in active infection (c), the maximal capacity of effector T cells is deployed by the immune system during primary infection in the attempt to avoid disease. Therefore, the number of initial memory T cells (and consequently the duration of protection) is further increased for 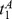, corresponding to the change in primary outcome from (b) latency to (c) active TB. For 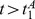, the poor response to generating memory T cells (decreasing 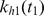) causes a monotonic decrease of the duration of immunity 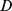.

**Figure 7:**
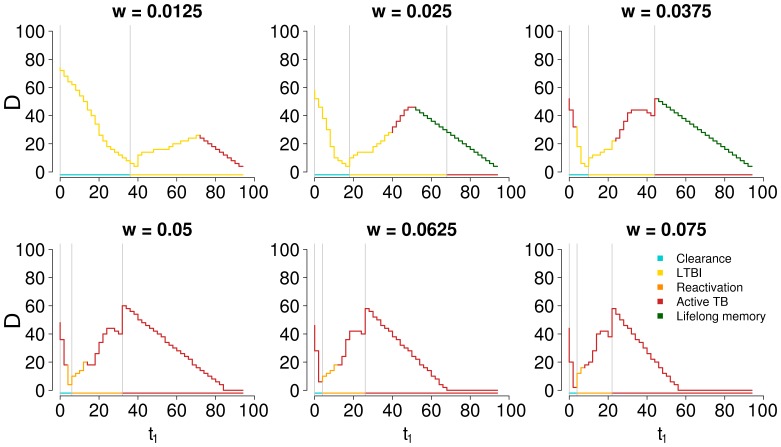
Duration of immunity D (years) by time of primary infection 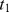 (years) and aging rate 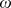 (years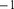). All graphs in the figure have the same x and y axis. The label for the y axis is reported only in the leftmost graph of each row. The label for the x axis is reported only in the bottom graph of each column. Analogous of Figure 6, recalculated with the CD4+ specific immunogenic potential 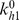 fixed to the baseline value of 0.1 ml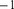. The curves are discrete because the times of primary and secondary infection have been sampled with a step of 2 years to reduce computational time.

Figure 6 shows how 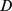 is nonlinearly dependent on 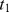; however, the effect of 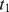 on immune dynamics will depend on the speed of the aging process, represented by the rate 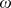. Therefore, Figure 7 shows the same graph of Figure 6, but using different values of the aging rate 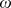. A similar pattern is found, but with a broader range of possible behaviors, due to variations with 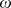 in primary infection outcome. By comparing panels, the effect of aging on the different parts of the duration curve can be more clearly isolated. In particular, the parts of the curve where aging is assumed to have a major role are regions (a) and (c): Figure 7 confirms that the negative slope of the curve in those regions gets more marked with increased values of 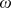.

### Key Mechanisms for the Generation of Memory

The key mechanisms for the generation of a robust pool of memory T cells, identified by sensitivity analysis, were found to be all positively correlated with the concentration of TNF-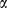 during primary response. Such mechanisms were the production of TNF-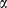 by CD4+ effector T cells and TNF-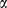 dependent chemotactic recruitment of both CD4+ effector T cells and macrophages (see Figure 3). Any other parameter significantly correlated with memory T cell numbers at 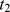 are strictly related to the intrinsical ability of the immune system to generate and maintain memory T cells, and not to the conditions of the primary immune response. Therefore, our model strongly supports the observation of a critical role played by TNF-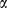 for the generation of a robust pool of memory T cells. This is in accordance with the link observed between anti-TNF treatment and reduced numbers of TB-specific memory T cells [66].

### Key Mechanisms for the Protective Effect of Memory Cells

As mentioned in the Introduction, the presence of memory T cells does not guarantee a favorable outcome upon secondary infection: the host’s immune system needs to be able to use such cells in an effective way. In the following lines, we use our model to analyze a number of aspects related to the efficacy of memory T cells in protecting the host from re-infection.

First, it has been proposed that the protection of resident memory T cells against secondary TB infection depends on the increased timeliness, rather than on the increased magnitude, of the immune response [67]. A rapid response could be due to the prompt deployment of effector function by resident memory T cells. In order to verify this hypothesis, we compared simulations in which an infection event occurred as primary (no memory), or as secondary with different initial values of resident memory T cells generated by a previous primary infection at varying age. We measured the time to peak and the peak numbers of CD4+ and CD8+ T cells for different values of the aging parameter in the model, both with and without memory. Table 2 summarizes these data, showing no difference in the magnitude of the response, and a significantly shorter time to peak for the model with memory. The model therefore lends support to the observations of [67].

**Table 2:** Comparison of timeliness and magnitude of models with and without memory.

Variable	Model with memory	Model without memory	units	p-value
average time to outcome	134.8	199.3	days	<10^−3^
peak CD4+ T cells	6011	5924	cells	0.25
peak CD8+ T cells	4964	4963	cells	0.50

Second, our model agrees with the common hypothesis that resident memory CD4+ T cells are more important than their CD8+ counterpart in protecting a host from exogenous re-infection [43], [44], [68], [69]. While the model in [50] predicted CD8+ T cells to be necessary for containment of primary infection, it also confirmed the relatively reduced contribution of resident memory CD8+ T cells against re-infection. In that study, clearance of secondary infection is obtained with very small numbers of initially available resident memory CD4+ T cells, compared to amounts required for resident memory CD8+ T cells. In this work, results from the sensitivity analysis confirm the conclusions of [50]: in fact, memory protection (indicated by target variable Q) is associated with higher values of the recruitment rate of CD4+ effector T cells during primary infection, but not correlated with any parameters related to CD8+ T cells (see Figure 3). In other words, increasing the number of resident memory CD4+ T cell (by an increased recruitment of effector cells during the acute phase) has a positive effect on protection; increasing the number of resident memory CD8+ T cell, instead, has no effect. It is also interesting to note that many parameters that are significant for inducing higher numbers of memory T cells at the time of re-infection (both CD4+ and CD8+) did not show up as significantly correlated with protection (see Figure 3): this confirms that the number of memory cells at re-infection is not the only determinant of protection.

Third, observations from murine systems [58], [59] indicate that resident memory T cells are sufficient for protection, ruling out the contribution of additional recruitment of circulating memory T cells to the site of infection. In accordance, no association was also found in the model between the rates of recruitment of circulating T cells to the lung, 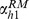 and 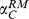, and the protective effect of memory as indicated by Q (Figure 3).

Thus, evidence suggests that protective memory is provided mainly by resident memory CD4+ cells, which rapidly initiate the immune response by means of their effector functions. The model fully agrees with this evidence. However, which specific effector functions are important for memory protection is still unclear. In a recent experiment [58], mice for which the production of IFN-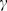 by CD4+ memory T cells had been blocked were found to be equally protected from exogenous reinfection with respect to fully functional mice. Therefore, it has been hypothesized that other functions of memory T cells [70], such as the production of TNF-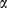, are responsible for protective immunity.

**Figure 8:**
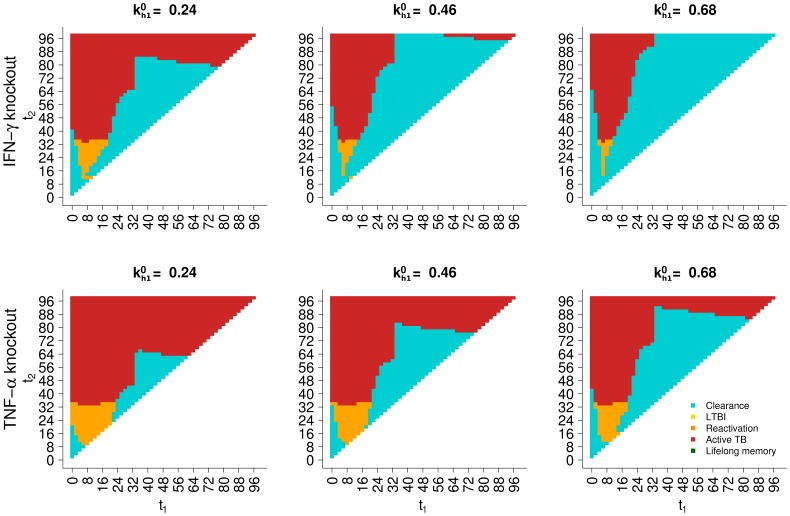
Simulated gene-knockout of cytokine production by memory T cells. All graphs in the figure have the same x and y axis. The label for the y axis is reported only in the leftmost graph of each row. The label for the x axis is reported only in the bottom graph of each column. Analogous of Figure 5, recalculated after knockout of cytokine production by memory T cells. Top row of panels: IFN-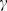 production knock-out. Bottom row of panels: TNF-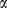 production knock-out. 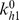: CD4+ specific immunogenic potential; 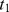: time of primary infection; 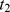: time of secondary infection. The graphs have a pixel-like appearance because the times of primary and secondary infection have been sampled with a step of 2 years to reduce computational time.

**Figure 9:**
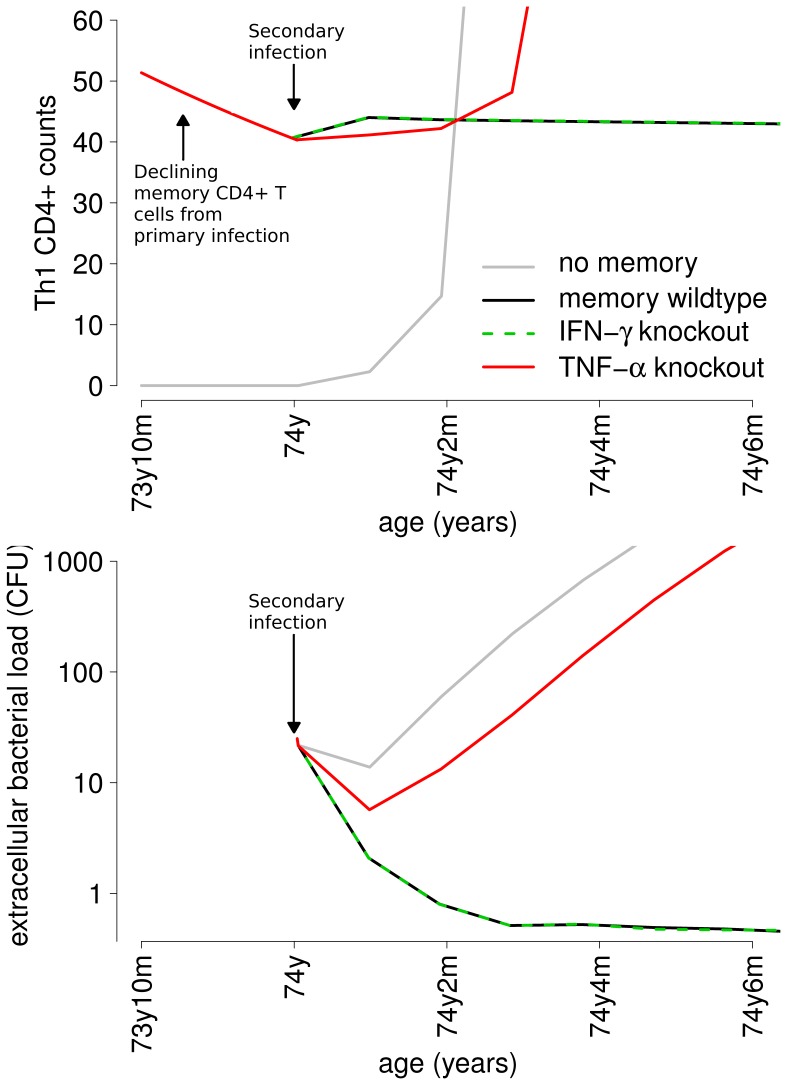
Timing of T cells in simulated gene-knockout experiments. Example of the evolution in time of the extracellular bacterial load and of T-helper type 1 (Th1) CD4+ effector T cells (both resident memory and non-memory) at secondary infection for CD4+ specific immunogenic potential 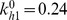 ml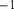, time of primary infection 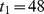 years and time of secondary infection 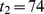 years.

To verify this hypothesis, we reproduced the simulations of Figure 5 under the conditions of an in-silico “knock-out” (deletion) as specified in the Materials and Methods section. We set to zero the levels and production by both CD4+ and CD8+ memory T cells of IFN-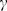 from the outset of the simulation, in one case, and of TNF-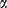, in another case. Results of these simulations are reported in Figure 8, where the top row of panels refers to IFN-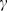 knock-out and the bottom row to TNF-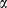 knock-out. Figure 8 confirms the observation in [58] that IFN-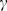 has virtually no role for protective immunity: in fact, the simulation outcomes are identical to those of the baseline (Figure 5). Furthermore, the importance of TNF-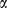 secretion by memory T cells is highlighted by the bottom row of Figure 8, where the regions of favorable outcomes are consistently reduced. Figure 9 shows that the protective effect of TNF-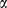 production by memory T cells is its ability to promptly recruit effector T cells to the site of re-infection. The top panel of Figure 9 reports Th1 CD4+ T cells, both memory and non-memory, as an example of changes in the immune response, while the bottom panel represents the extracellular bacterial load. In these simulations, secondary infection occurs at age 74 years, when the initial pool of memory CD4+ T cells produced at primary infection (at age 48 years, not shown) is declining in time (left side of graph in the top panel; the lines for the baseline, IFN-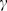 knockout and TNF-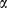 knockout scenarios overlap for 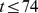 years). At secondary infection, the extracellular bacterial load inoculated decreases in the first month of infection in all scenarios (bottom panel), due to the intervention of adaptive immune response, as can be seen in the top panel by the increase of CD4+ T cells. Such increase in T cells (decrease in bacterial load) is initially less rapid for the TNF-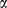 knock-out simulation (red lines) than for the baseline (black lines) and the IFN-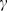 knock-out scenarios (green dashed lines). The delay in the availability of cytotoxic T cells in the TNF-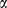 knockout and in the no-memory scenario allows Mtb to survive the initial immune response (bottom panel) and eventually overcome it, leading to active disease. In the baseline and IFN-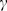 knock-out scenarios, instead, the initial abundance of CD4+ T cells is sufficient to keep Mtb under control and clear the infection. In summary, the model identifies TNF-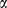 production by resident memory CD4+ T cells as a major determinant of protection from reinfection, supporting an hypothesis which has not been investigated before in wet labs.

## Discussion

In this work we studied the role of aging and memory in the dynamics of TB infection. A more comprehensive understanding of these mechanisms is much needed in order to identify better treatments against age-related endogenous reactivation of latent TB infection and to develop highly immunogenic vaccines. The proposed model suggests answers to related questions in TB research: i) what factors are responsible for the increased reactivation risk in older ages? ii) what are the determinants of a strong memory response upon primary infection? iii) what is the role of cytokines in effective memory response against a secondary infection? The model is validated by its ability to qualitatively reproduce several features of endogenous reactivation and of protective immunity against exogenous re-infection.

Considering immunosenescence in the dynamics of immune response to primary TB infection has a two-fold effect. First, age determines the ability of the immune system to control the pathogen during the initial phase of the response, which is critical for the outcome of primary infection (clearance vs. LTBI vs. active disease). In fact, an older age at primary infection implies a lower value of the age-dependent CD4+ T cells recruitment parameters, and therefore a delayed availability of effector CD4+ T cells, thus increasing the chances of active TB, as previously found in aged mice [54]. Second, when infection is controlled in a latent state, aging contributes to endogenous reactivation by altering the dynamic equilibrium between bacterial growth and the bactericidal activity of the immune system, established in the acute phase of the primary response. The model therefore predicts that, as the replacement of lost CD4+ T cells is slowed due to aging recruitment processes, the capacity of the immune system to control bacteria is impaired. The model also suggests that endogenous reactivation occurs via a threshold mechanism with respect to the recruitment rates of CD4+ T cells, with reactivation occurring at the same age regardless of the time of primary infection.

Even though immunosenescence has cumulative adverse effects, the memory response can help offset some of them. The availability of resident memory T cells at the time of secondary infection can prevent the host from developing active TB. This is something that has been postulated in epidemiological models of TB infection [55].

Immune protection induced by memory depends on several factors, among which are the timing of primary and secondary infections in the life of the host. This dependence operates according to different mechanisms. The amount of memory T cells initially generated depends on both the immunogenic potential of the host against the pathogen and on the number of effector T cells produced during primary acute response. According to the model, in older individuals a more adverse primary infection (characterized by higher numbers of effector T cells) results in a positive effect on the duration of memory protection even if the immunogenic potential is weakened due to immunosenescence. This prediction reflects data from [71], where the size of the memory pool was found to be highest in patients with mild active TB, intermediate in LTBI (measured via Tuberculin Skin Test (TST) ) and lowest in cleared or unexposed hosts (negative TST). Furthermore, the interval between primary and secondary infection episodes determines how much of the initial memory pool has waned at the time of secondary infection.

Clearly, human hosts are able to clear most viral and bacterial infections on their own, sometimes aided by antibiotics or antivirals. However, this is not the case for persistent infections such as TB, chickenpox, or typhoid fever. Thus, prevention becomes as much of a critical issue as treatment in these diseases. Our model of persistent infection studying the role of aging and memory indicates recruitment of CD4+ T cells to the site of infection as a fundamental process to target in order to affect a significant change in the long term dynamics of persistent infections. If recruitment is augmented in latently infected hosts, endogenous reactivation events can be slowed down or avoided, reducing the probability of active disease.

In addition, the model supports the concept that high TNF-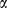 levels during primary infection are associated with increased strength and duration of memory protection. More in general, enhancing the recruitment of CD4+ T cells during natural infection or immunization with potential vaccines could result in a longer lasting protective effect of memory. We believe that this result could have important implications for the development of vaccine adjuvants for TB [72]. Overall, strategies targeted to recruitment mechanisms, both towards preventive treatment of latent infection and immunization, could have a significant impact on the clinical outcome for infected hosts and on the global burden of disease.

Finally, *in silico* experiments performed in this study suggest that TNF-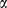 production by resident memory CD4+ T cells during secondary response is determinant for protection, supporting an hypothesis [70] that has been only proposed but not tested yet; wet lab experiments similar to those performed on other cytokines [58] could provide compelling evidence towards this hypothesis.

The main limitation of this study is a general lack of immunological data from human subjects that would be needed both to estimate model parameters and to quantitatively validate the model. The lack of data is compounded by the long time scales involved in immunosenescence and memory mechanisms requiring longitudinal within-host studies. We have compensated for this lack of data by comparing model predictions with a wide set of diverse qualitative observations from human and animal studies, and through the use of uncertainty and sensitivity analysis. This technique served both to quantify the effects of parameter uncertainty on model outcomes and to identify critical nodes of the system. Thus, our results are successfully interpreted in a qualitative framework.

Many other complex aspects of TB immunology have been simplified in this first attempt to model lifelong interactions between Mtb and human hosts. The model does not take into account the impact of immunological changes occurring in human hosts during their transition from childhood to adulthood. These changes are presumably responsible for the increased vulnerability of children to infections, and for the different manifestation of TB disease in children (primarily extra-pulmonary vs. primarily pulmonary in adults) and are not considered here. Our goal here is to study adult pulmonary TB.

In addition, modeling spatial aspects of the immune response to Mtb at the time scale of a host’s life might provide additional useful insight on aging and memory, due to the critical role of spatial dynamics in granuloma formation [49], [8], [5]. However, available models of granuloma formation cannot be easily simulated over temporal scales longer than one year, because they are based on a technique (Agent Based Modeling) which is computationally intensive even in high performance computing architectures. On the other hand, the reference model [50] on which the present work is based summarizes well the outcome of TB infection even without including spatial dynamics, thus representing a good trade-off between computational feasibility and model appropriateness.

Finally, memory studies require knowledge of and data for specific antigens. In TB there are multiple antigens that have been identified as immunogenic, however no single one has been pinpointed as the epitope responsible for the generation of memory in TB and this remains an open question. In summary, our study is theoretical in nature, but it is able to lend key insights in the dynamics of host response to Mtb infection.

## Supporting Information

File S1 (PDF)
