# Supplementary material for: The Roles of Immune Memory and Aging in Protective Immunity and Endogenous Reactivation of Tuberculosis
Source: PLoS One. 2013 Apr 8;8(4):e60425. doi: 10.1371/journal.pone.0060425 (PMC3620273; doi:10.1371/journal.pone.0060425)
Supplement: File S1 — (PDF) [file pone.0060425.s001.pdf]

Supporting Information

The roles of immune memory and aging in protective immunity and endogenous reactivation of tuberculosis

Giorgio Guzzetta[1,2,*], Denise Kirschner[3]
1 Department of Statistics and Mathematics Applied to Economics, University of Pisa, Italy
2 Fondazione Bruno Kessler, Trento, Italy
3 Department of Microbiology and Immunology, University of Michigan Medical School, USA * E-mail: guzzetta@fbk.eu

S1 Equations and compartmental scheme of the reference model

Hereafter we report the Ordinary Differential Equations for the reference model of acute immune response [1] on which the present work builds, with each contributing term shortly defined on the right side. Additional information on biological support for the specific functional form of each can be found in the original paper [1]. A graphical summary of these dynamics for cellular populations are represented in Figure S1.

$\frac{dM_R}{dt} =$	**RESTING MACROPHAGES** M_R
$+Sr_m$	Background recruitment
$+\alpha_{4a}(M_A + w_2 M_I)$	Macrophage recruitment
$+Sr_{4b}\frac{F_\alpha}{F_\alpha+f_8 I_{10}+s_{4b}}$	TNF-α recruitment
$-k_2 M_R \frac{BE}{BE+c_9}$	Infection (to M_I)
$-k_3 M_R \frac{BE+wBI+\beta F_\alpha}{BE+wBI+\beta F_\alpha+c_8}\frac{I_\gamma}{I_\gamma+f_1 I_4+s_1}$	Activation (to M_A)
$-\nu_{MR} M_R$	Natural death
$\frac{dM_I}{dt} =$	**INFECTED MACROPHAGES** M_I
$+k_2 M_R \frac{BE}{BE+c_9}$	Infection (from M_R)
$-k_{17} M_I \frac{BI^2}{BI^2+N^2 MI^2}$	Bursting
$-k_{14a} M_I \frac{\frac{T_C+w_3 T_{h1}}{M_I}}{\frac{T_C+w_3 T_I}{M_I}+c_4}$	T cell induced apoptosis
$-k_{14b} M_I \frac{F_\alpha}{F_\alpha+f_9 I_{10}+s_{4b}}$	TNF-α induced apoptosis
$-k_{52} M_I \frac{\frac{T_C\frac{T_{h1}}{T_{h1}+c_{T1}}+w_1 T_{h1}}{M_I}}{\frac{T_C\frac{T_{h1}}{T_{h1}+c_{T1}}+w_1 T_{h1}}{M_I+c_{52}}}$	Cytotoxic killing by T cells
$-\nu_{MI} M_I$	Natural death
$\frac{dM_A}{dt} =$	**ACTIVATED MACROPHAGES** M_A
$+k_3 M_R \frac{BE+wBI+\beta F_\alpha}{BE+wBI+\beta F_\alpha+c_8}\frac{I_\gamma}{I_\gamma+f_1 I_4+s_1}$	Activation (from M_R)
$-k_4 M_A \frac{I_{10}}{I_{10}+s_8}$	Deactivation
$-\nu_{MA} M_A$	Natural death

$\frac{dT_0}{dt}$ = **INDIFFERENTIATED CD4 T CELLS** T_0

$+\alpha_{1a}(M_A + w_2 M_I)$	Macrophage recruitment
$+Sr_{1b}\frac{F_\alpha}{F_\alpha+f_8 I_{10}+s_{4b2}}$	TNF-α recruitment
$+\alpha_2 T_0 \frac{M_A}{M_A+c_{15}}$	Proliferation
$-k_6 I_{12} T_0 \frac{I_\gamma}{I_\gamma+f_1 I_4+f_7 I_{10}+s_1}$	Differentiation (to T_{h1})
$-k_7 T_0 \frac{I_4}{I_4+f_2 I_\gamma+s_2}$	Differentiation (to T_{h2})
$-\nu_{T0} T_0$	Natural death

$\frac{dT_{h1}}{dt}$ = **T-HELPER TYPE 1 CD4+ CELLS** T_{h1}

$+\alpha_{3a}(M_A + w_2 M_I)$	Macrophage recruitment
$+Sr_{3b}\frac{F_\alpha}{F_\alpha+f_8 I_{10}+s_{4b1}}$	TNF-α recruitment
$+k_6 I_{12} T_0 \frac{I_\gamma}{I_\gamma+f_1 I_4+f_7 I_{10}+s_1}$	Differentiation (from T_0)
$-\nu_{Tg}\frac{I_\gamma}{I_\gamma+c} T_{h1} M_A$	IFN-γ apoptosis
$-\nu_{T1} T_{h1}$	Natural death

$\frac{dT_{h2}}{dt}$ = **T-HELPER TYPE 2 CD4+ CELLS** T_{h2}

$+\alpha_{3a2}(M_A + w_2 M_I)$	Macrophage recruitment
$+Sr_{3b2}\frac{F_\alpha}{F_\alpha+f_8 I_{10}+s_{4b1}}$	TNF-α recruitment
$+k_7 T_0 \frac{I_4}{I_4+f_2 I_\gamma+s_2}$	Differentiation (from T_0)
$-\nu_{T2} T_{h2}$	Natural death

$\frac{dT_{80}}{dt}$ = **INDIFFERENTIATED CD8 T CELLS** T_{80}

$+\alpha_{1a}(M_A + w_2 M_I)$	Macrophage recruitment
$+Sr_{1b}\frac{F_\alpha}{F_\alpha+f_8 I_{10}+s_{4b2}}$	TNF-α recruitment
$+\alpha_2 T_{80} \frac{M_A}{M_A+c_{15}}$	Proliferation
$-k_6 I_{12} T_{80} \frac{I_\gamma}{I_\gamma+f_1 I_4+f_7 I_{10}+s_1}$	Differentiation (to T_C and T_8)
$-\nu_{T0} T_{80}$	Natural death

$\frac{dT_C}{dt}$ = **CD8+ T CELLS WITH CYTOTOXIC FUNCTION** T_C

$+m\alpha_{3ac}(M_A + w_2 M_I)$	Macrophage recruitment
$+mSr_{3bc}\frac{F_\alpha}{F_\alpha+f_8 I_{10}+s_{4b1}}$	TNF-α recruitment
$+mk_6 I_{12} T_{80} \frac{I_\gamma}{I_\gamma+f_1 I_4+f_7 I_{10}+s_1}$	Differentiation (from T_{80})
$-\nu_{TCg}\frac{I_\gamma}{I_\gamma+c_c} T_C M_A$	IFN-γ apoptosis
$-\nu_{TC} T_C$	Natural death

$\frac{dT_8}{dt}$ = **CD8+ T CELLS** T_8

$+m\alpha_{3ac}(M_A + w_2 M_I)$	Macrophages recruitment
$+mSr_{3bc}\frac{F_\alpha}{F_\alpha+f_8 I_{10}+s_{4b1}}$	TNF-α recruitment
$+mk_6 I_{12} T_{80} \frac{I_\gamma}{I_\gamma+f_1 I_4+f_7 I_{10}+s_1}$	Differentiation (from T_{80})
$-\nu_{TCg}\frac{I_\gamma}{I_\gamma+c_c} T_8 M_A$	IFN-γ apoptosis
$-\nu_{TC} T_8$	Natural death

$\frac{dBE}{dt} =$	**EXTRACELLULAR BACTERIA** BE
$+\alpha_{20}BE$	Bacterial replication
$+k_{17}NM_I\frac{BI^2}{BI^2+N^2M_I^2}$	Bursting of M_I (from BI)
$+k_{14a}NN_{fracc}M_I\frac{\frac{T_C+w_3T_{h1}}{M_I}}{\frac{T_C+w_3T_{h1}}{M_I+c_4}}$	T cell induced apoptosis of M_I (from BI)
$+k_{14b}NN_{fraca}M_I\frac{F_\alpha}{F_\alpha+f_9I_{10}+s_{4b}}$	TNF-α induced apoptosis of M_I (from BI)
$-k_2\frac{N}{2}M_R\frac{BE}{BE+c_9}$	Internalization by M_R (to BI)
$-k_{15}M_ABE - k_{18}M_RBE$	Killing by macrophages
$-n_EBE$	Natural death

$\frac{dBI}{dt} =$	**INTRACELLULAR BACTERIA** BI
$+\alpha_{19}BI(1-\frac{BI^2}{BI^2+N^2M_I^2})$	Bacterial replication
$+k_2\frac{N}{2}M_R\frac{BE}{BE+c_9}$	Internalization by M_R (from BE)
$-k_{17}NM_I\frac{BI^2}{BI^2+N^2M_I^2}$	Bursting of M_I (to BE)
$-k_{14a}NM_I\frac{\frac{T_c+w_3T_{h1}}{M_I}}{\frac{T_c+w_3T_{h1}}{M_I+c_4}}$	T cell induced apoptosis of M_I (partially to BE)
$-k_{14b}NM_I\frac{F_\alpha}{F_\alpha+f_9I_{10}+s_{4b}}$	TNF-α induced apoptosis of M_I (partially to BE)
$-k_{52}NM_I\frac{\frac{T_C\frac{T_{h1}}{T_{h1}+c_{T1}}+w_1T_{h1}}{M_I}}{\frac{T_C\frac{T_{h1}}{T_{h1}+c_{T1}}+w_1T_{h1}}{M_I+c_{52}}}$	Cytotoxic killing of M_I by T cells
$-n_IBI$	Natural death

$\frac{dI_\gamma}{dt}$	$=$		**INTERFERON-γ I_γ**
		$+s_g \frac{BE+wBI}{BE+wBI+c_{10}} \frac{I_{12}}{I_{12}+s_7}$	Dendritic cells production
		$+\alpha_{5a} T_{h1} \frac{M_A}{M_A+c_{5a}}$	T_{h1} production
		$+\alpha_7 T_0 \frac{I_{12}}{I_{12}+f_4 I_{10}+s_4}$	T_0 production
		$+\alpha_7 T_{80} \frac{I_{12}}{I_{12}+f_4 I_{10}+s_4}$	T_{80} production
		$+\alpha_{5b} T_8 \frac{M_A}{M_A+c_{5b}}$	T_8 production
		$+\alpha_{5c} M_I$	M_I production
		$-\nu_{I\gamma} I_\gamma$	Natural decay
$\frac{dI_{12}}{dt}$	$=$		**INTERLEUKIN-12 I_{12}**
		$+s_{12} \frac{BE+wBI}{BE+wBI+c_{230}}$	Dendritic cells production
		$+\alpha_{23} M_R \frac{BE+wBI}{BE+wBI+c_{23}}$	M_R production
		$+\alpha_8 M_A \frac{s}{s+I_{10}}$	M_A production
		$-\nu_{I12} I_{12}$	Natural decay
$\frac{dI_{10}}{dt}$	$=$		**INTERLEUKIN-10 I_{10}**
		$+\delta_7 M_I \frac{s_6}{I_{10}+f_6 I_\gamma+s_6}$	M_I production
		$+\alpha_{16} T_{h1}$	T_{h1} production
		$+\alpha_{17} T_{h2}$	T_{h2} production
		$+\alpha_{18} \frac{T_C+T_8}{2m}$	CD8+ T cells production
		$-\nu_{I10} I_{10}$	Natural decay
$\frac{dI_4}{dt}$	$=$		**INTERLEUKIN-4 I_4**
		$+\alpha_{11} T_0$	T_0 production
		$+\alpha_{12} T_{h2}$	T_2 production
		$-\nu_{I4} I_4$	Natural decay
$\frac{dF_\alpha}{dt}$	$=$		**TUMOR NECROSIS FACTOR-α F_α**
		$+\alpha_{30} M_I$	M_I production
		$+\alpha_{31} M_A \frac{I_\gamma+\beta_2(BE+wBI)}{I_\gamma+\beta_2(BE+wBI)+f_1 I_4+s_{10}}$	M_A production
		$+\alpha_{32} T_{h1}$	T_{h1} production
		$+\alpha_{33} \frac{T_C+T_8}{2m}$	CD8+ T cells production
		$-\nu_{F\alpha} F_\alpha$	Natural decay

S2 Derivation of Equations for the Immune Memory Generation and Maintenance

For the dynamics of the circulating memory T cells compartment (T^{CM}), we refer to a well established mathematical model of memory generation [2]. This model assumes that an initial amount of memory T cells is generated at the site of primary infection during the contraction phase of the immune response. This amount is slowly diluted during the lifetime of the individual as a consequence of several factors, including the reduction of thymic input of naive T cells and the competition of memory T cells specific for different antigens within the immune memory pool [2]. These dynamics are very complex (reviewed in [3, 4, 5]) and their modeling goes beyond the scope of this work. Therefore we assume the following equation for T^{CM}:

$$T_i^{CM}(t) = k_i T_i^{peak} e^{-d(t-t_1)} \tag{1}$$

where $i = h1, C$ distinguishes CD4+ from CD8+ T cells, T^{peak} is the peak number of effector T cells during the acute phase of primary infection, k_i is the fraction of effector T cells which are initially transformed to circulating memory T cell and d is the rate at which the pool of circulating memory T cells wanes in time due to dilution, assumed constant and equal for both CD4+ and CD8+ T cells.

Dynamics of resident memory T cells can be modeled in terms of a balance between the natural death of T cells and the antigen-independent recruitment of T cells from the circulating memory compartment [6]:

$$\frac{dT_i^{RM}}{dt} = sr_i^{RM} T_i^{CM}(t) - \mu_i^{RM} T_i^{RM}(t)$$

Given that changes in the value of $T_i^{CM}(t)$ occur in the time scale of years or decades, whereas the dynamics of recruitment and death of T_i^{RM} are in the time scale of days [6], we can approximate $T_i^{CM}(t)$ as constant in time. Therefore, at equilibrium we will have:

$$T_i^{RM} = \frac{sr_i^{RM}}{\mu_i^{RM}} T_i^{CM}(t) = h_i^{RM} T_i^{CM}(t) \tag{2}$$

In other words, the amount of resident memory T cells at any time is approximated as a proportion h_i^{RM} of the concentration of circulating memory T cells and therefore this number wanes in time with the same rate d as the circulating memory compartment.

S3 Estimation of Parameter Values and Ranges

Most of the model parameters used throughout this work were estimated in previous published papers [7, 8, 9, 1] and are reported in Table S3.

In order to estimate the 8 parameter values and ranges for the additional equations reported in the previous section, human data from published studies on TB-specific memory lymphocytes are used where possible.

Estimation of k_i

Using Equation 1, k_i can be estimated for $t = t_1$ as

$$k_i = \frac{T_i^{CM}(t_1)}{T_i^{peak}}$$

Experimental studies [16] found about 20 (range: 5 to 250) central memory CD4+ T cells every 10^6 Peripheral Blood Mononuclear Cells (PBMC) in the blood of TB patients 6 months after successful treatment. In another study [17] about 300 (range: 200 to 700) central memory CD8+ T cells every 10^6 PBMC were detected in the blood of TB patients 4 months after treatment. We indicate these quantities as f_4^{CM} and f_8^{CM} respectively. Assuming that the peak of the acute phase of the immune response had been reached by the time at which f_i^{CM} were measured, we can obtain the concentration $T_i^{CM}(t_1)$ of memory T cells in the blood by simple multiplication of f_i^{CM} by the normal lymphocyte concentration in the blood [18], indicated with $[PBMC]$. Range and average values for T_i^{peak} were estimated by simulating a primary infection with 1,000 different parameter sets, chosen by Latin Hypercube Sampling [19] using uniform distributions within their range of variability.

Table S2 summarizes the process of estimation for k_i.

Estimation of h_{RMi}

h_i^{RM} can be estimated from Equation 2 given measurements on resident memory T cells and estimates of circulating memory T cells (as already provided in Table S2) at $t = t_1$. In an experimental study on individuals with LTBI (ascertained by reactivity to Tuberculin Skin Test) [20], about 750,000 CD4+ and 350,000 CD8+ were found on average in the broncho-alveolar lavage (BAL) fluid of patients (volume $V_{BAL} = 30$cc); 7.4 to 14% of CD4+ (55,000 to 105,000) and about 0.8% of CD8+ (3,000) expressed an effector memory phenotype (production of IFN-γ and other inflammatory cytokines) after 2 days from a new intranasal challenge with Protein Purified Derivative (PPD) proteins. Therefore, these cells can be considered resident memory T cells: we term the corresponding quantities $t_{h1}^{RM}(t_1)$ and $t_C^{RM}(t_1)$ respectively. Since the site of infection considered in our model is a cube of alveolar parenchima of size $V_{site} = 2\text{x}2\text{x}2\text{mm} = 8 \cdot 10^{-3}$ cc, we can estimate the number $T_i^{RM}(t_1)$ of initial resident memory T cells at the site of a secondary infection as:

$$T_i^{RM}(t_1) = t_i^{RM}(t_1)\frac{V_{site}}{V_{BAL}}$$

and h_i^{RM} as $\frac{T_i^{RM}(t_1)}{T_i^{CM}(t_1)}$. These passages are summarized in Table S3.

Estimation of α_i^{CM}

The products $\alpha_i^{CM} T_i^{CM}$ represent the macrophage-dependent recruitment rate of circulating memory T cells, in analogy to parameters α_{3a} and α_{3ac} in the terms describing the macrophage-dependent recruitment of effector T cells in the reference model [1]. We assume that the recruitment of memory T cells from the circulating compartment can be κ times that of the macrophage-dependent term, with κ broadly chosen between 0.1 and 100. Table S4 reports a summary of the estimation process for the ranges of these parameters.

Estimation of ω and d

ω is the rate of the exponential decay of CD4+ T cell recruitment occurring due to immunosenescence, while d is the rate at which memory T cells decay due to mechanisms of immune memory dilution [2]. Since we could not find any data to estimate these parameters, we choose a broad range of possible values in order to include all possible ranges of behavior. Values for both rates were chosen between 0.01 and 0.1 years^{-1}, in such a way that a host may lose 50% of his initial capacity (pool of circulating memory T cells for d, and CD4+ T cells recruitment rates for ω) in about 7 to 70 years. These numbers take as an approximate reference the time to equilibrium of a primary TB infection (up to 5 years) and the maximal lifetime of an individual (about 100 years). We choose for both rates a baseline value of 0.05 years^{-1}. This corresponds to a half-life of about 15 years, which is a good reference for both d (corresponding to the estimated duration of memory protection from Bacillus Calmette Guerin (BCG) vaccination [21]) and ω (age at which, according to [22], the risk of primary active disease is intermediate between children of age ≤ 10 years and adults ≥ 20 years).

S4 Endogenous reactivation

The immune response model with aging is able to reproduce endogenous reactivation in a broad region of the parameter space. Figure S2 displays the final outcome of infection for several different values of three model parameters: the rate of cytotoxic killing by activated CD8+ T cells, k_{52}, which selects the panel in Figure S2; the initial value of the TNF-α dependent recruitment rate of CD4+ cells, Sr_{3b}^0 (x-axis); and the aging parameter ω (y-axis). These parameters were selected because of their importance on model

outcome after a preliminary sensitivity analysis [1]. The outcome can be one of four possible types: a) active disease, displayed in dark red; b) LTBI followed by endogenous reactivation at some point of the host's life (orange); c) LTBI without reactivation (yellow); d) clearance of primary infection (turquoise).

References

1. Sud D, Bigbee C, Flynn JAL, Kirschner DE (2006) Contribution of CD8 T Cells to control of Mycobacterium tuberculosis infection. The Journal of Immunology 176: 4296–4314.

2. Antia R, Pilyugin SS, Ahmed R (1998) Models of immune memory: On the role of cross-reactive stimulation, competition, and homeostasis in maintaining immune memory. PNAS 95: 14926–14931.

3. Nikolich-Zugich J (2008) Ageing and life-long maintenance of T-cell subsets in the face of latent persistent infections. Nature Reviews Immunology 8: 512–522.

4. Miller RA (2005) T cells in aging mice: genetic, developmental, and biochemical analysis. Immunological Reviews 205: 94–103.

5. Buchholz VR, Neuenhahn M, Busch DH (2011) CD8+ T cell differentiation in the aging immune system: until the last clone standing. Current Opinion in Immunology 23: 1–6.

6. Ely KH, Cookenham T, Roberts AD, Woodland DL (2006) Memory T Cell Populations in the Lung Airways Are Maintained by Continual Recruitment. The Journal of Immunology 176: 537–543.

7. Wigginton JE, Kirschner DE (2001) A Model to Predict Cell-Mediated Immune Regulatory Mechanisms During Human Infection with Mycobacterium tuberculosis. The Journal of Immunology 166: 1951–1967.

8. Marino S, Pawar S, Fuller CL, Reinhart TA, Flynn JL, et al. (2004) Dendritic Cell Trafficking and Antigen Presentation in the Human Immune Response to Mycobacterium tuberculosis. The Journal of Immunology 173: 494–506.

9. Chang ST, Linderman JJ, Kirschner DE (2005) Multiple mechanisms allow Mycobacterium tuberculosis to continuously inhibit MHC class II-mediated antigen presentation by macrophages. PNAS 102: 4530–4535.

10. Gilbertson B, Zhong J, Cheers C (1999) Anergy, IFN-γ production, and apoptosis in terminal infection of mice with Mycobacterium avium. J Immunol 163: 2073–2080.

11. Tsukaguchi K, de Lange B, Boom WH (1999) Differential regulation of IFN-γ, TNF-α, and IL-10 production by CD4(+) $\alpha\beta$ TCR+ T cells and Vδ2(+)$\gamma\delta$ T cells in response to monocytes infected with Mycobacterium tuberculosis-H37Ra. Cell Immunol 194: 12–20.

12. Oddo M, Renno T, Attinger A, Bakker T, MacDonald HR, et al. (1998) Fas ligand-induced apoptosis of infected human macrophages reduces the viability of intracellular Mycobacterium tuberculosis. J Immunol 160: 5448–5454.

13. Rojas M, Olivier M, Gros P, Barrera LF, Garcia LF (1999) TNF-α and IL-10 modulate the induction of apoptosis by virulent Mycobacterium tuberculosis in murine macrophages. J Immunol 162: 6122–6131.

14. Li L, Sad S, Kagi D, Mosmann TR (1997) CD8Tc1 and Tc2 cells secrete distinct cytokine patterns in vitro and in vivo but induce similar inflammatory reactions. J Immunol 158: 4152–4161.

15. DAmico G, Frascaroli G, Bianchi G, Transidico P, Doni A, et al. (2000) Uncoupling of inflammatory chemokine receptors by IL-10: generation of functional decoys. Nat Immunol 1: 387–391.

16. Millington KA, Innes JA, Hackforth S, Hinks TSC, Deeks JJ, et al. (2007) Dynamic Relationship between IFN-gamma and IL-2 Profile of Mycobacterium tuberculosis-Specific T Cells and Antigen Load. The Journal of Immunology 178: 5217–5226.

17. Caccamo N, Guggino G, Meraviglia S, Gelsomino G, Di Carlo P, et al. (2009) Analysis of Mycobacterium tuberculosis-Specific CD8 TCells in Patients with Active Tuberculosis and in Individuals with Latent Infection. PLoS ONE 4: e5528.

18. McClatchey KD (2001) Clinical laboratory medicine. Lippincott Williams & Wilkins.

19. Marino S, Hogue IB, Ray CJ, Kirschner DE (2008) A Methodology For Performing Global Uncertainty and Sensitivity Analysis in Systems Biology. Journal of Theoretical Biology 254: 178–196.

20. Walrath J, Zukowski L, Krywiak A, Silver RF (2005) Resident Th1-Like Effector Memory Cells in Pulmonary Recall Responses to Mycobacterium tuberculosis. Am J Respir Cell Mol Biol 33: 48–55.

21. Styblo K, Meijer J (1976) Impact of BCG vaccination programmes in children and young adults on the tuberculosis problem. Tubercle.

22. Vynnycky E, Fine PEM (1997) The natural history of tuberculosis: the implications of age-dependent risks of disease and the role of reinfection. Epidemiology and Infection 119: 183–201.

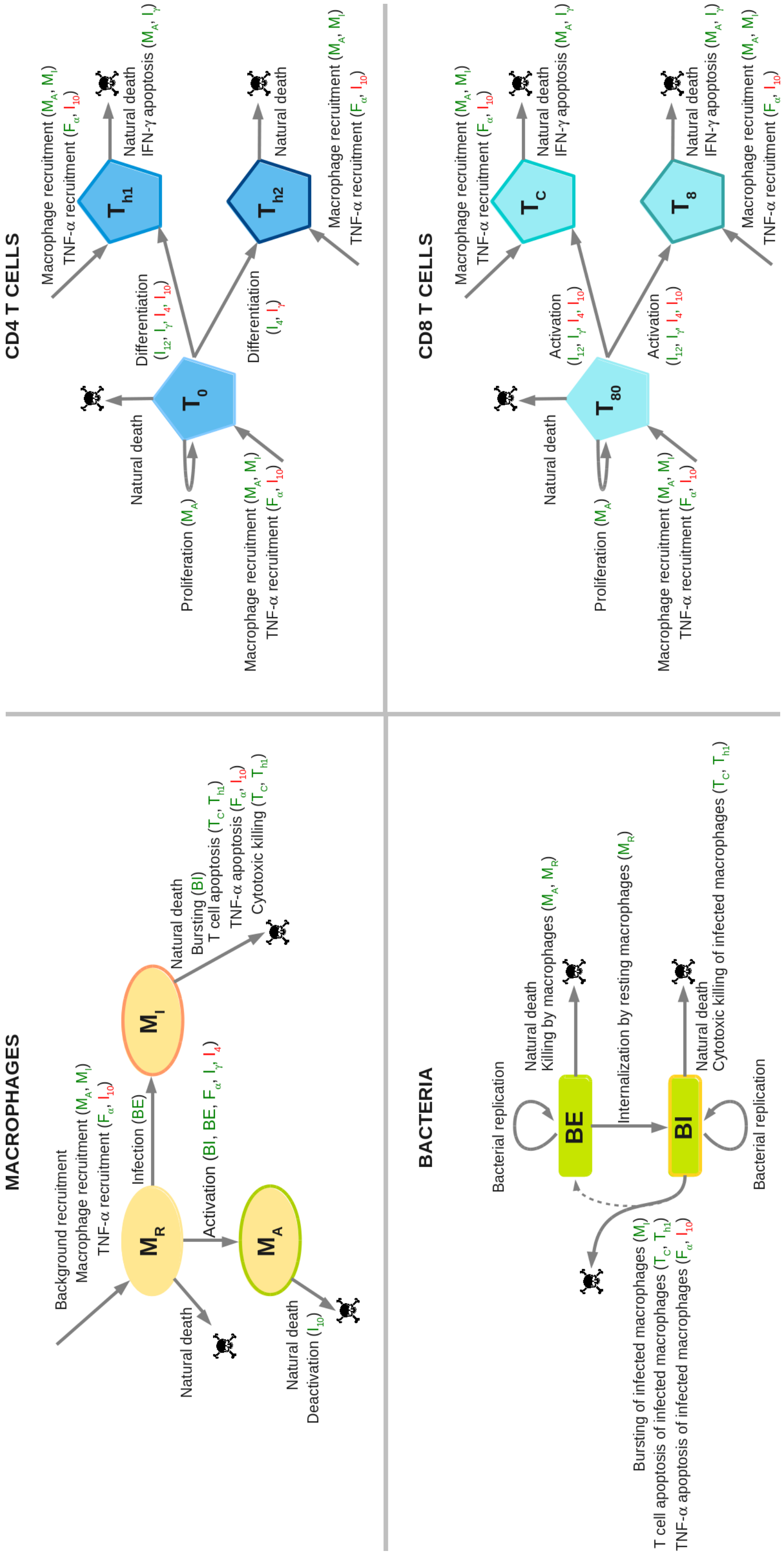

Figure S1: **Graphical summary of the main interactions of the immune response to Mtb.** Each arrow represents a change of state for each cellular population; text alongside arrows describes the terms contributing to each change of state; variables listed in parentheses after each term description represent cells and cytokines enhancing (green), or inhibiting (red) the given mechanism.

Table S1: **Parameter ranges and baseline values for model parameters.**

**Parameter name**	**Symbol**	**Ref.**	**Min**	**Base**	**Max**	**Unit**
M_R recruitment rate	Sr_m	[7]	600	650	700	M_R/day
Contribution of BI to M_R activation	w	[7]	0.01	0.1	0.5	-
Contribution of M_I to M_R recruitment	w_2	[1]	0.0005	0.05	0.5	-
Ratio adjustment I_{10}/F_α on M_R recruitment	f_8	[10]	1	60	100	-
Half saturation of F_α onr M_R recruitment	s_{4b}	[10]	100	200	556	pg/ml
Half saturation of F_α on T_0 recruitment	s_{4b2}	[10]	100	200	500	pg/ml
Half saturation of I_{10} on M_A deactivation	s_8	[7]	100	1000	2000	pg/ml
Half saturation of BE on M_R infection	c_9	[7]	10^5	$5 \cdot 10^6$	$3 \cdot 10^7$	pg/ml
Adjustment I_4/I_γ	f_1	[7]	2.9	205	410	-
Scaling factor of F_α for M_R activation	β	[1]	100	1000	10^5	1/pg
M_R death rate	ν_{MR}	[7]	0.0033	0.0033	0.0033	1/day
Carrying capacity of M_I	N	[7]	40	75	100	-
Half saturation of T_{h1}/M_I ratio on M_I apoptosis	c_4	[7]	1	40	60	-
Cytotoxic killing of M_I	k_{52}	[1]	0.07	0.2	1	1/day
Half saturation of T_C on M_I killing	c_{52}	[1]	10	20	100	-
M_I death rate	ν_{MI}	[7]	0.0011	0.0011	0.0011	1/day
Macrophage recruitment of T_0	α_{1a}	[11]	0.001	0.003	0.01	1/day
Max growth rate of T_0	α_2	[7]	0.01	2	2.8	1/day
Max T_0 to T_{h1} rate	k_6	[7]	0.005	0.005	0.005	ml/(pg da
Max T_0 to T_{h2} rate	k_7	[7]	0.02	0.03	0.07	ml/(pg da
Half saturation I_4	s_2	[7]	1	1.5	2	pg/ml
Percentage overlap between T_C and T_8	m	[1]	0.5	0.7	1	-
F_α dependent recruitment of T_{h1}	Sr^0_{3b}	[11]	10^4	$5 \cdot 10^4$	10^5	1/day
F_α dependent recruitment of T_{h2}	Sr_{3b2}	[11]	1000	10^5	10^5	1/day
Half saturation I_γ on T_{h1} apoptosis	c	[12]	1067	1120	1173	pg/ml
T_{h2} death rate	ν_{T2}	[7]	0.3	0.3	0.3	1/day
F_α dependent recruitment of T_C and T_8	Sr_{3bc}	[11]	10^4	$5 \cdot 10^4$	$8 \cdot 10^4$	1/day
Half saturation I_γ on T_C and T_8 apoptosis	c_c	[12]	530	550	600	pg/ml
I_γ production by dendritic cells (DCs)	s_g	[7]	1	500	1000	pg/(ml da
Half saturation of I_{12} on I_γ production by DCs	s_7	[7]	5	60	100	pg/ml
Half saturation of M_A on I_γ production by T_{h1}	c_{5a}	[1]	5000	8000	20000	1/ml
I_γ production by M_I	α_{5c}	[1]	0.02	0.05	0.066	pg/ml
I_γ production by T_0	α_7	[7]	0.02	0.05	0.066	pg/ml
Half saturation of I_{12} on I_γ	s_4	[7]	50	75	100	pg/ml
I_γ decay rate	ν_{IG}	[7]	2.16	20	33.27	1/day
Half saturation of Mtb on I_{12} production by M_R	c_{23}	[1]	1000	10^5	$5 \cdot 10^6$	1/ml
Dendritic cell production of I_{12}	s_{12}	[1]	200	500	1000	pg/(ml da
I_12 decay rate	ν_{I12}	[7]	1.1	1.1	1.1	1/day
I_{12} production by M_A	α_8	[7]	0.0012	0.005	0.012	pg/day
Adjustment I_γ on I_{10}	f_6	[7]	0.025	0.035	0.053	-
I_{10} production by T_{h1}	α_{16}	[7]	$2 \cdot 10^{-4}$	$8 \cdot 10^{-4}$	10^{-3}	pg/day
I_{10} production by T_C and T_8	α_{18}	[1]	$2 \cdot 10^{-4}$	$4 \cdot 10^{-3}$	$6 \cdot 10^{-2}$	pg/day
I_{10} decay rate	ν_{I10}	[7]	3.7	5.5	7.23	1/day
I_4 production by T_{h2}	α_{12}	[7]	0.001	0.0065	0.00912	pg/day
F_α production by M_I	α_{30}	[1]	0.0012	0.012	0.02	pg/(ml da
Scaling factor of Mtb for F_α production by M_A	β_2	[1]	10^{-3}	10^{-3}	10^{-3}	1/pg
F_α production by T_{h1}	α_{32}	[13]	10^{-5}	10^{-4}	$5 \cdot 10^{-3}$	pg/(ml da
Half saturation of Mtb on F_α production by T_{h1}	c_T	[1]	1000	5000	10000	-
BI growth rate	α_{19}	[7]	0.1	0.35	1	1/day
Fraction BI released by T cell apoptosis of M_I	N_{fracc}	[7]	0.05	0.1	0.2	-
BE killing by M_A	k_{15}	[7]	$1.3 \cdot 10^{-9}$	$1.25 \cdot 10^{-6}$	$1.25 \cdot 10^{-5}$	ml/day
BI death rate	n_I	[1]	$5 \cdot 10^{-6}$	$5 \cdot 10^{-3}$	$5 \cdot 10^{-3}$	1/day
Macrophage recruitment of M_R	α_{4a}	[1]	0.01	0.03	0.05	1/day
Max contribution of T_{h1} to M_I apoptosis	w_3	[1]	0.1	0.4	0.8	-
F_α dependent recruitment of M_R	Sr_{4b}	[1]	$2 \cdot 10^3$	$2 \cdot 10^5$	$5 \cdot 10^5$	1/day
Ratio adjustment F_α/I_{10}	f_9	[1]	1	60	100	-

**Parameter name**	**Symbol**	**Ref.**	**Min**	**Base**	**Max**	**Unit**
Half saturation of F_α dependent T_{h1} recruitment	s_{4b1}	[1]	160	168	172	pg/ml
M_A deactivation by I_{10}	k_4	[7]	0.01	0.36	0.40	1/day
M_R infection rate	k_2	[7]	0.01	0.05	5	1/day
M_R activation rate	k_3	[7]	0.001	0.01	0.1	1/day
Half saturation of I_γ dependent M_R activation	s_1	[7]	0.1	10	30	pg/ml
Half saturation of BE and BI on M_R activation	c_8	[7]	10^4	10^5	$2.2 \cdot 10^5$	1/ml
Max rate of M_I bursting	k_{17}	[7]	$8 \cdot 10^{-5}$	0.05	0.08	1/day
T cell induced apoptosis of M_I	k_{14a}	[1]	0.03	0.05	0.08	1/day
F_α induced apoptosis of M_I	k_{14b}	[1]	0.15	0.5	1.00	1/day
Max contribution of T_{h1} to cytotoxic killing	w_1	[1]	0.001	0.5	1.00	-
Half saturation of T_{h1} on cytotoxic killing	c_{T1}	[1]	1	50	10000	-
M_A death rate	ν_{MA}	[7]	0.07	0.07	0.07	1/day
$F_/alpha$ dependent T_0 recruitment	Sr_{1b}	[11]	10^4	10^5	10^6	1/day
Half saturation of M_A on I_γ production by T_{h1}	c_{15}	[7]	10^4	$5 \cdot 10^4$	10^5	-
Effect of I_{10} on I_γ induced differentiation of T_0 to T_{h1}	f_7	[1]	1	5	5.00	-
Adjustment I_γ/I_4	f_2	[7]	1	1	1	-
T_0 death rate	ν_{T0}	[7]	0.0111	0.2	0.333	1/day
Macrophage recruitment of T_{h1}	α^0_{3a}	[11]	0.003	0.003	0.003	1/day
Macrophage recruitment of T_{h2}	α_{3a2}	[1]	10^{-3}	10^{-3}	10^{-3}	1/day
I_γ induced apoptosis of T_{h1}	ν_{Tg}	[12]	10^{-5}	10^{-4}	10^{-3}	1/day
T_{h1} death rate	ν_{T1}	[7]	0.3	0.3	0.30	1/day
Macrophage recruitment of T_C and T_8	α_{3ac}	[11]	0.001	0.0077	0.0145	1/day
I_γ induced apoptosis of T_c and T_8	ν_{TCg}	[12]	10^{-5}	10^{-4}	10^{-3}	1/day
T_C death rate	ν_{TC}	[1]	0.3	0.3	0.3	1/day
Half saturation of Mtb on I_γ production by DCs	c_{10}	[7]	100	5000	10^5	1/ml
I_γ production by T_{h1}	α_{5a}	[1]	0.01	0.5	1	pg/day
I_γ production by T_8	α_{5b}	[1]	0.01	0.5	1	pg/day
Half saturation of M_A on I_γ production by T_8	c_{5b}	[1]	10^4	$5 \cdot 10^4$	10^5	1/ml
Adjustment of I_{10}/I_{12} on I_γ	f_4	[7]	0.76	1.8	3.20	-
Half saturation of M_A on I_γ production by T_8	c_{5b}	[1]	10^4	$5 \cdot 10^4$	10^5	1/ml
I_{12} production by M_R	α_{23}	[1]	10^{-4}	10^{-3}	0.1	pg/ml
I_{12} production by M_A	α_8	[7]	10^{-4}	0.003	0.01	pg/day
Half saturation of Mtb on I_{12} production by DCs	c_{230}	[1]	1000	10^4	10^5	1/ml
I_{10} effect on I_{12} production by M_A	s	[1]	1	50	100	pg/ml
Half saturation of I_{10} self-inhibition in M_A	s_6	[7]	51	56	60	pg/ml
I_{10} production by M_A	δ_7	[1]	10^{-5}	10^{-2}	0.1	pg/ml
I_{10} production by T_{h2}	α_{17}	[7]	$2 \cdot 10^{-4}$	$6 \cdot 10^{-3}$	$6 \cdot 10^{-3}$	pg/day
I_4 production by T_0	α_{11}	[7]	0.001	0.002	0.004	pg/day
I_4 decay rate	ν_{I4}	[7]	2.77	2.77	2.77	1/day
F_α production by M_A	α_{31}	[1]	0.003	0.01	0.015	pg/(ml da
Half saturation of I_γ on F_α production by M_A	s_{10}	[1]	50	80	100	pg/ml
F_α production by T_8	α_{33}	[14]	$6.4 \cdot 10^{-5}$	10^{-4}	$1.1 \cdot 10^{-4}$	pg/(ml da
F_α decay rate	ν_{TNF}	[15]	1.112	1.112	1.112	1/day
BE growth rate	α_{20}	[7]	0.05	0.05	0.05	
Fraction BI released by TNF apoptosis of M_I	N_{fraca}	[1]	0.4	0.6	0.8	-
BE killing by M_R	k_{18}	[7]	$1.3 \cdot 10^{-9}$	$2 \cdot 10^{-9}$	$7 \cdot 10^{-8}$	ml/day

Table S2: **Estimation of the immunogenic potentials, k_i.**

**Parameter name**	**Symbol**	**Reference**	**Min**	**Max**	**Mean**	**Unit**
Proportion of T_{h1}^{CM} cells in blood	f_{h1}^{CM}	[16]	5	250	20	$\frac{cells}{10^6 PBMC}$
Proportion of T_C^{CM} in blood	[17]	f_C^{CM}	200	700	300	$\frac{cells}{10^6 PBMC}$
Lymphocyte concentration in blood	$[PBMC]$	[18]	$1.5 \cdot 10^6$	$5 \cdot 10^6$	$3 \cdot 10^6$	$\frac{PBMC}{ml}$
Concentration of T_{h1}^{CM} in blood	$T_{h1}^{CM}(t_1)$	$f_{h1}^{CM} \cdot [PBMC]$	7.5	1250	60	$\frac{cells}{ml}$
Concentration of T_C^{CM} in blood	$T_C^{CM}(t_1)$	$f_C^{CM} \cdot [PBMC]$	300	3500	900	$\frac{cells}{ml}$
Number of peak T_{h1} cells	T_{h1}^{peak}	[1]	30	20000	600	$cells$
Number of peak T_C cells	T_C^{peak}	[1]	30	15000	600	$cells$
Immunogenic potential for T_{h1}^{CM}	k_{h1}	$\frac{T_{h1}^{CM}(t_1)}{T_{h1}^{peak}}$	0.0625	0.25	0.1	ml^{-1}
Immunogenic potential for T_C^{CM}	k_C	$\frac{T_C^{CM}(t_1)}{T_C^{peak}}$	0.23	10	1.5	ml^{-1}

Table S3: **Estimation of the turnover rates of resident memory T cells, h_i^{RM}.**

**Parameter name**	**Symbol**	**Reference**	**Min**	**Max**	**Mean**	**Unit**
Volume of BAL	V_{BAL}	[20]	-	-	30	cc
Volume of site of infection	V_{site}	[1]	-	-	$8 \cdot 10^{-3}$	cc
Initial number of T_{h1}^{RM} in BAL	$t_{h1}^{RM}(t_1)$	[20]	55,000	105,000	80,000	$cells$
Initial number of T_C^{RM} in BAL	$t_C^{RM}(t_1)$	[20]	3000	3000	3000	$cells$
Initial number of T_{h1}^{RM}	$T_{h1}^{RM}(t_1)$	$t_{h1}^{RM}(t_1)\frac{V_{site}}{V_{BAL}}$	15	28	21	$cells$
Initial number of T_C^{RM}	$T_C^{RM}(t_1)$	$t_C^{RM}(t_1)\frac{V_{site}}{V_{BAL}}$	1	1	1	$cells$
Concentration of initial T_{h1}^{CM}	$T_{h1}^{CM}(t_1)$	Table S2	7.5	1250	60	$\frac{cells}{ml}$
Concentration of initial T_C^{CM}	$T_C^{CM}(t_1)$	Table S2	300	3500	900	$\frac{cells}{ml}$
Turnover rate of memory CD4+	h_{h1}^{RM}	$\frac{T_{h1}^{RM}(t_1)}{T_{h1}^{CM}(t_1)}$	2.24 10-2	2	0.35	ml
Turnover rate of memory CD8+	h_C^{RM}	$\frac{T_C^{RM}(t_1)}{T_C^{CM}(t_1)}$	2.9 10-4	3.3 10-3	1.1 10-3	ml

Table S4: **Estimation of the antigen dependent recruitment rates of circulating memory T cells upon secondary infection, α_i^{CM}.**

**Parameter name**	**Symbol**	**Reference**	**Min**	**Max**	**Mean**	**Unit**
Arbitrary proportional constant	κ	-	0.1	100	1	
Initial concentration of T_{h1}^{CM}	$T_{h1}^{CM}(t_1)$	Table S2	7.5	1250	60	$\frac{cells}{ml}$
Initial concentration of T_C^{CM}	$T_C^{CM}(t_1)$	Table S2	300	3500	900	$\frac{cells}{ml}$
Rate of T_{h1} recruitment by macrophages	α_{3a}	[1]	0.003	0.003	0.003	$\frac{cells/Mph}{day}$
Rate of T_C recruitment by macrophages	α_{3ac}	[1]	0.003	0.003	0.003	$\frac{cells/Mph}{day}$
T_{h1}^{CM} recruitment rate	α_{h1}^{CM}	$\kappa\frac{\alpha_{3a}}{T_{h1}^{CM}(t_1)}$	$2.4 \cdot 10^{-7}$	0.04	$5 \cdot 10^{-5}$	$\frac{ml/Mph}{day}$
T_C^{CM}	α_C^{CM}	$\kappa\frac{\alpha_{3ac}}{T_C^{CM}(t_1)}$	$8.6 \cdot 10^{-8}$	10^{-3}	$3.3 \cdot 10^{-6}$	$\frac{ml/Mph}{day}$

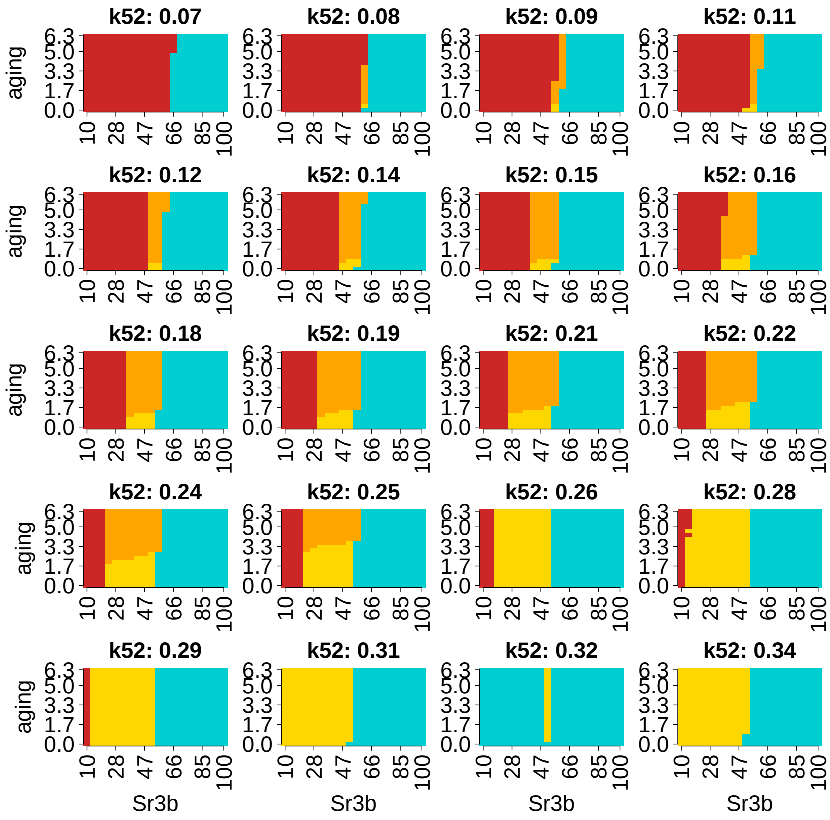

Figure S2: **Parameter regions for reactivation.** All simulations are performed assuming an initial inoculum of $N = 25$ bacteria, the age at first infection $t_1 = 0$ for simplicity, and all other model parameters as in Table S3 of this text.
